# Author Correction: Class I HDAC inhibitors enhance YB-1 acetylation and oxidative stress to block sarcoma metastasis

**DOI:** 10.1038/s44319-025-00478-6

**Published:** 2025-05-29

**Authors:** Amal M El-Naggar, Syam Prakash Somasekharan, Yemin Wang, Hongwei Cheng, Gian Luca Negri, Melvin Pan, Xue Qi Wang, Alberto Delaidelli, Bo Rafn, Jordan Cran, Fan Zhang, Haifeng Zhang, Shane Colborne, Martin Gleave, Anna Mandinova, Nancy Kedersha, Christopher S Hughes, Didier Surdez, Olivier Delattre, Yuzhuo Wang, David G Huntsman, Gregg B Morin, Poul H Sorensen

**Affiliations:** 1 Department of Pathology & Laboratory Medicine, University of British Columbia, Vancouver, BC V6T 2B5 Canada; 2 Department of Molecular Oncology, BC Cancer, part of the Provincial Health Services Authority, Vancouver, BC V5Z1L3 Canada; 3 Department of Pathology, Faculty of Medicine, Menoufia University, Shibin Al Kawm, Egypt; 4 Vancouver Prostate Centre, Vancouver, BC V5Z 1M9 Canada; 5 Michael Smith Genome Sciences Centre, Vancouver, BC V5Z4S6 Canada; 6 Brigham and Women’s Hospital, Harvard University, Boston, MA 02115 USA; 7 Massachusetts General Hospital, Harvard University, Boston, MA 02114 USA; 8 Centre de recherche de l’Institut Curie, 75005 Paris, France

## Abstract

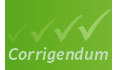

**Correction to:** *EMBO Reports* (2019) 20:e48375. 10.15252/embr.201948375 | Published online 31 October 2019

The journal contacted the authors after becoming aware of a potential image aberration in the paper. Based on the exchanges and data provided by the authors, the journal retracts and replaces the following figure panels. Corresponding original images are published with this notice:

**Figure 5A is retracted and replaced**.

**Figure 5A:**
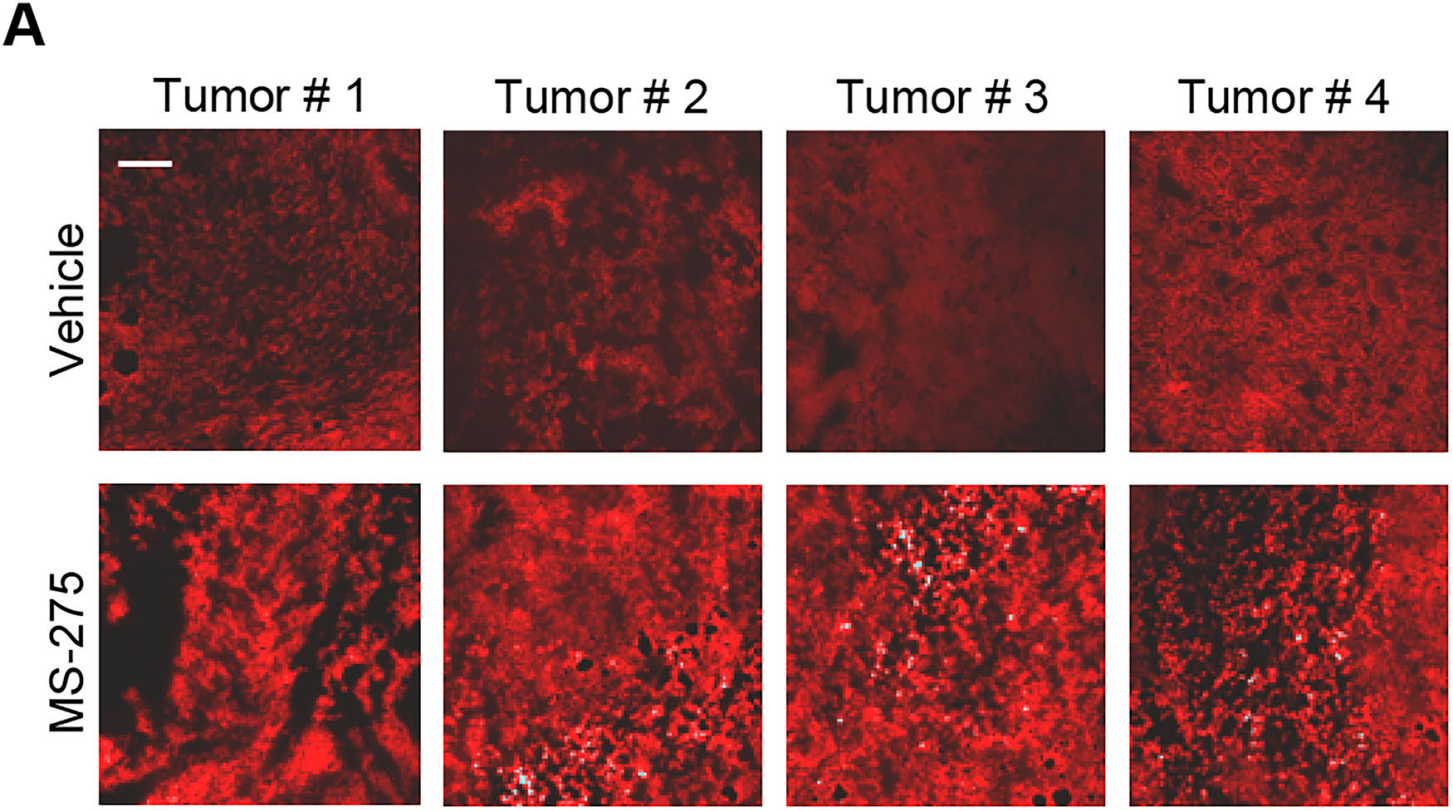
**Original.**

**Figure 5A:**
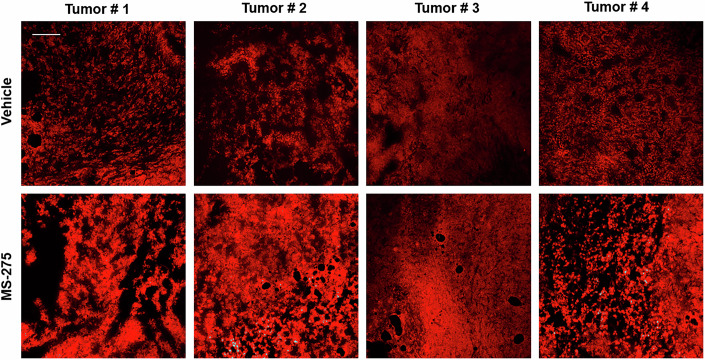
Corrected. Source data are available online for this figure.

**Figure 5A source data is published with this correction**.

Author statement:

We identified an error in Figure 5A where an image from tumor T2 was mistakenly placed under tumor T3 (both from the MS-275 group). This error occurred during the selection of images from .ppt files used for publication. Importantly, this mistake does not affect data analysis, as the original images were correctly stored and analyzed separately for each tumor. The error occurred within the same treatment group and was an inadvertent oversight.

This author correction does not affect the conclusions of the manuscript.

A.M.El-Naggar, S.P.Somasekharan, Y.Wang, G.L.Negri, M. Pan, X.Q.Wang, A.Delaidelli, B.Rafn, J.Cran, F.Zhang, H.Zhang, M.Gleave, A.Mandinova, C.S.Hughes, O.Delattre, Y.Wang, D.G.Huntsman, G.B.Morin and P.H.Sorensen agree to this author correction. No response could be obtained from H.Cheng, N.Kedersha, S.Colborne and D.Surdez.

## Supplementary information

Figure 5A Source Data

