# Supplementary material for: Class I HDAC inhibitors enhance YB‐1 acetylation and oxidative stress to block sarcoma metastasis
Source: EMBO Rep. 2019 Oct 31;20(12):e48375. doi: 10.15252/embr.201948375 (PMC6893361; doi:10.15252/embr.201948375)
Supplement: Supplementary file 1 — Appendix [file EMBR-20-e48375-s001.pdf]

Table of Contents

APPENDIX TABLE S1 2

APPENDIX TABLE S2 3

APPENDIX TABLE S1

Compounds significantly enhanced $NaAsO_2$-induced death in U2OS cells

**Compound**	**Function**
**MS-275**	Selective inhibitor of class I histone deacetylases (HDACs)
**CAY10603**	HDAC6 selective inhibitor
**5B939**	Pan-HDAC inhibitor (no effect on HDAC6)
**SAHA**	Pan-HDAC inhibitor
**Phthalazinone pyrazole**	Aurora A inhibitor
**JQ1**	BRD4 inhibitors
**Quisinostat**	Selective inhibitor of Class I and II HDACs
**Panobinostat**	Non-selective histone deacetylase inhibitor
**Romidepsin**	Selective inhibitor of class I HDACs

APPENDIX TABLE S2

KEY RESOURCES

**REAGENT**	**SOURCE**	**Cat#**
**Antibodies**		
4- HNE	Abcam	ab46545
Agarose-conjugated anti-acetyllysine antibody	Immunechem Inc.	ICP0388
Ac-HC: rabbit polyclonal	Millipore Corp	Cat # 06-866
Akt (total): rabbit polyclonal	NEB Canada	Cat # 9272
β-actin: rabbit polyclonal	Cell Signaling Technology	Cat # D6A8
Caspase-3: rabbit polyclonal	Cell Signaling Technology	Cat # 9662
Cleaved caspase-3: rabbit polyclonal	Cell Signaling Technology	Cat # 9661
Cleaved PARP: mouse monoclonal	BD Biosciences	Cat # 552596
CD99: rabbit polyclonal	Abcam	Cat # Ab27271
FLAG: mouse monoclonal	Sigma-Aldrich	Cat # F1804-1MG
FMRP: mouse monoclonal	Santa Cruz	Cat # sc-101048
G3BP1: mouse monoclonal	Novus Biologicals	Cat # NBP2-16563
G3BP: mouse monoclonal	BD Transductions	Cat # 611126
GAPDH: rabbit monoclonal	Cell Signaling Technology	Cat # 2118S
GRB2: mouse monoclonal	BD Transductions	Cat # 610111

Histone H4 antibody: rabbit polyclonal	Abcam	Cat # Ab61255
Ki67	Thermo Scientific	Cat # RM-9106
NRF2: rabbit polyclonal	Cell Signaling Technology	Cat # 12721S
NRF2: rabbit polyclonal	Abcam	Cat # ab62352
NRF2: mouse monoclonal	Santa Cruz	Cat # sc-365949
NRF2 (phosphor S40): rabbit monoclonal	Abcam	Cat # ab76026
PARP: rabbit polyclonal	Cell Signaling Technology	Cat # 9542S
TIA-1: rabbit polyclonal	Abcam	Cat # ab40693
YB-1 (D299): rabbit polyclonal	Cell Signaling Technology	Cat # 4202
YB-1 (59-Q): mouse monoclonal	Santa Cruz	Cat # sc-101198
Alexa Fluor 488	Invitrogen	Cat # A11017
Alexa Fluor 594	Invitrogen	Cat # A11012
Normal mouse IgG	Santa Cruz	Cat # sc-2025
Normal rabbit IgG	Cell Signaling Technology	Cat # 2729S
Osteosarcoma TMA	Biomax	Cat # OS804b
**Chemicals, Peptides, and Recombinant Proteins**		
Sodium arsenite Solution ($NaASO_2$)	Sigma-Aldrich	Cat # 70039
Pierce ECL Western Blotting Substrate	Thermo Fisher Scientific	Cat # 32106
Recombinant YB-1 protein	Abnova	Cat # H00004904-P01

Reagent or Resource		
Epigenetic screening library	Cayman	Cat # 11076
RNeasy Plus Universal Kit	Qiagen	Cat # 73404
High-Capacity cDNA Reverse Transcription kit	Applied Biosystems	Cat # 4368813
Fast SYBR Green Master Mix	Thermo Fisher Scientific	Cat # 4385616
Custom DMEM	Caisson Laboratories	Cat # DML04
Dialyzed fetal bovine serum (dFBS)	Life Technologies	Cat # 26400-036
4x LDS Sample buffer	ThermoFisher	Cat # 84788
Anti-FLAG® M2 Magnetic Beads	Sigma- Aldrich	Cat # M8823-5ML
Streptavidin M-280 beads	Life Technologies	Ct # 11205D
Protein A/G magnetic beads	Pierce Biotechnology Inc/Fisher	Cat # PI88802
Azidohomoalanine (AHA)	Anaspec	Cat # 63669
Antifade Mounting Medium with DAPI	Vector Laboratories	Cat # H1200
Biotin alkyne	Invitrogen	Cat # B10185
Click-iT Protein Reaction Buffer kit	Invitrogen	Cat # C10276
CM-H2DCFDA	Invitrogen	Cat # C6827
Draq5	Cedarlane	Cat # DR50050
Halt Protease and Phosphatase Inhibitor Cocktail, EDTA-free (100X)	Thermo Fisher Scientific	Cat # 78441

Paraformaldehyde 32% Solution (EM Grade)	Cedarlane	Cat # 15714-S
siLentFect Transfection Reagent	Bio-Rad	Cat # 1703360
Entinostat (MS-275)	Cedarlane (Selleckchem)	Cat # S1053
**Critical Commercial Assays**		
GSH/GSSG-Glo™ Assay	Promega	Cat # V6611
ARE Reporter Kit	BPS Bioscience	Cat # 60514
Quick-start Bradford assay	Bio-Rad	Cat # 500-0205
**Oligonucleotides**		
ACTB<br><br>F: 5'-TCCCCCAACTTGAGATGTATG-3'<br><br>R: 5'-ACTGGTCTCAAGTCAGTGTACAGG -3'	IDT	N/A
G3BP1<br><br>F: 5'- TGAGGTCTTTGGTGGGTTTG -3'<br><br>R: 5'- TGCTGTCTTTCTTCAGGTTCC -3'	IDT	N/A
GAPDH<br><br>F: 5'- ACCCACTCCTCCACCTTTGA -3'<br><br>R: 5'- CTGTTGCTGTAGCCAAATTCGT -3'	IDT	N/A
HIF1A<br><br>F: 5'-TGATGACCAGCAACTTGAGG-3'<br><br>R: 5'-CTGGGGCATGGTAAAAGAAA-3'	IDT	N/A

YBX1<br><br>F: 5'- GGAGGGTGCTGACAACCA -3'<br><br>R: 5'- GCTGTCTTTGGCGAGGAG -3'	IDT	N/A
NFE2L2<br><br>F: 5'- CAGATGCCACAGTCAACACA -3'<br><br>R: 5'- GGGCTCAGCTATGAAAGCA -3'	IDT	N/A
