# Supplementary material for: Class I HDAC inhibitors enhance YB‐1 acetylation and oxidative stress to block sarcoma metastasis
Source: EMBO Rep. 2019 Oct 31;20(12):e48375. doi: 10.15252/embr.201948375 (PMC6893361; doi:10.15252/embr.201948375)
Supplement: Supplementary file 2 — Expanded View Figures PDF [file EMBR-20-e48375-s002.pdf]

Expanded View Figures

Figure EV1. Increased ROS production in 3D culture of sarcoma cells.

A Effects of MS-275 treatment at the indicated doses on U2OS cell proliferation as detected by Incucyte. The graph represents fold change relative to day 0, with error bar = standard deviation for *n* = 3 independent experiments.

B U2OS cells treated +/− MS-275 and $NaAsO_2$ (100 μM, 1 h) were assessed for ROS levels using CM-H_2DCFDA. ROS levels were normalized to protein content. Error bars indicate SEM for *n* = 3 independent experiments, each performed in triplicate.

C CHLA-10 cells were treated without (vehicle) or with MS-275 (1 μM, 24 h), and without (−$NaAsO_2$) or with $NaAsO_2$ (+$NaAsO_2$) (500 μM, 1 h) and assessed for ROS levels using CM-H_2DCFDA. Data are presented as fold change over vehicle control. Error bars indicate SEM for *n* = 3 independent experiments; each performed in triplicate

D U2OS cells treated +/− MS-275 and $NaAsO_2$ were assessed for ROS levels using the fluorogenic probe, CellRox. Data are presented as fold change over vehicle control. Error bars indicate SEM for *n* = 3 independent experiments; each performed in triplicate.

E U2OS cells grown in monolayer cultures (2D) were treated with vehicle alone or antioxidants NAC (5 mM) and catalase (2,000 units/ml), prior to transfer to new 2D plates or ultra-low attachment surface plates (3D) and cultured for a further 18 h with continuation of treatment as indicated. ROS levels were then assessed using CM-H_2DCFDA. Data are presented as fold change over vehicle control. Error bars indicate SEM for *n* = 3 independent experiments; each performed in triplicate.

F U2OS cells grown in monolayers (3D−) or ultra-low attachment plates (3D+) +/− MS-275 and the antioxidant NAC were assessed for ROS levels using CM-H_2DCFDA. Data are presented as fold change over vehicle control. Error bars indicate SEM for *n* = 3 independent experiments; each performed in triplicate.

G Effects of antioxidants on the viability of U2OS cells grown in 3D cultures in the presence of MS-275 as above, determined by immunoblotting for cleaved caspase-3 as indicated. GRB2 was used as a loading control.

H Fractionation of polysomes extracted from U2OS cells +/− MS-275 (1 μM, 24 h), and +/− $NaAsO_2$ (100 μM, 1 h) on sucrose gradients displays marked differences in ribosome patterns dependent on displayed treatments. Total cytoplasmic polysomes were separated on sucrose gradients (10–45%) prepared using a BioComp Gradient Station unit and centrifuged using an SW60Ti rotor. Gradients were fractionated using a BioComp Gradient Fractionator equipped with a Triax module for acquisition of UV signal (260 nm).

I, J Top panels: Immunoblots showing NRF2 protein decay after cycloheximide (CHX) addition in U2OS (I) and CHLA-10 (J) cells +/− MS-275 treatment. Cells were pre-treated with the NRF2 inducing agent, SFN (L-sulforaphane) at 20 μM conc for 4 h. Then, CHX was added along with SFN for the indicated time periods. GAPDH was used loading controls. Bottom panels: Graphical representation of NRF2 protein levels based on densitometry in U2OS (top panel I) and CHLA-10 (top panel J) cells +/− MS-275 treatment at the indicated time points after cycloheximide (CHX). Half-lives are shown under the curves, representing results of two independent experiments ± SEM.

Data information: Unpaired two-tailed Student's *t*-test; *$P < 0.05$; **$P < 0.005$; ***$P < 0.0005$; n.s = non-significant.
Source data are available online for this figure.

© 2019 The Authors

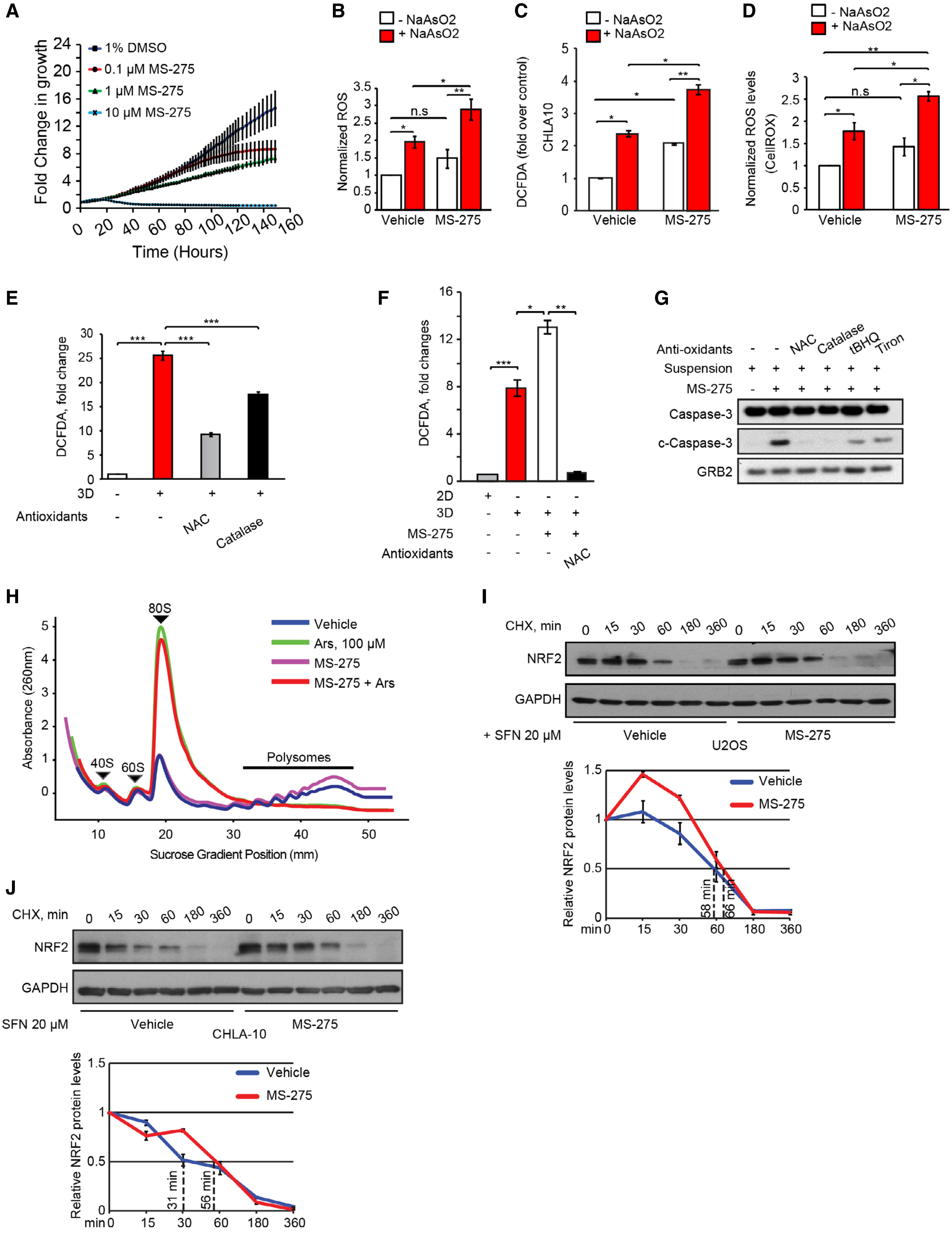
A
1% DMSO
0.1 μM MS-275
1 μM MS-275
10 μM MS-275
Fold Change in growth
Time (Hours)
B
- NaAsO2
+ NaAsO2
Normalized ROS
Vehicle MS-275
C
DCFDA (fold over control) CHLA10
D
Normalized ROS levels (CellROX)
E
DCFDA, fold change
3D
Antioxidants
NAC
Catalase
F
DCFDA, fold changes
2D
3D
MS-275
Antioxidants
NAC
G
Anti-oxidants
Suspension
MS-275
NAC Catalase tBHQ Tiron
Caspase-3
c-Caspase-3
GRB2
H
80S
40S
60S
Polysomes
Vehicle
Ars, 100 μM
MS-275
MS-275 + Ars
Absorbance (260nm)
Sucrose Gradient Position (mm)
I
CHX, min
NRF2
GAPDH
+ SFN 20 μM
Vehicle
MS-275
U2OS
Relative NRF2 protein levels
58 min
66 min
J
CHX, min
NRF2
GAPDH
+ SFN 20 μM
Vehicle
MS-275
CHLA-10
Relative NRF2 protein levels
31 min
56 min

Figure EV1.

Figure EV2. MS-275-induced acetylation of translation-associated proteins.

A Heatmap of translation-associated proteins and histones from SILAC analysis, showing fold change represented as log2 transformed ratios of heavy amino acid (MS-275 treated; +MS-275) over light amino acid (vehicle; −MS-275) conditions with or without $NaAsO_2$ treatment (500 μM, 1 h), as described in the Materials and Methods. Color scale represents Log2 SILAC Ratio heavy/light (H/L).

B Analysis of YB-1 and G3BP1 acetylation in U2OS cells +/− MS-275 (1 μM, 24 h) and $NaAsO_2$ (500 μM, 1 h) as indicated. Lysine-acetylated proteins were affinity purified using anti-acetyl-lysine (α-acK) antibodies and analyzed by immunoblotting, using antibodies to YB-1 or G3BP. IgG was used as negative antibody control and GRB2 as a loading control.

C Immunoblotting showing time course analysis of Ac-H4 (top) and total histone H4 acetylation (middle) in response to MS-275 treatment (1 μM, 24 h) in U2OS cells. IgG was used as negative antibody control and GAPDH as a loading control.

D Analysis of YB-1 acetylation in U2OS cells +/− the indicated class I HDAC inhibitor treatments for 24 h; MS-275 (1 μM), Quisinostat, and Romidepsin (100 nM). Lysine-acetylated proteins were affinity purified using anti-acetyl-lysine (α-acK) antibodies and analyzed by immunoblotting, using antibodies to YB-1. IgG was used as negative antibody control and GAPDH as a loading control.

E Immunoblotting showing expression levels of FLAG-tagged wt YB-1 or the indicated lysine (K) to alanine (A) (K-to-A) mutant (K81A) (top arrow) or endogenous YB-1 in U2OS cells, as analyzed by antibodies to YB-1. GRB2 was used as a loading control.

Source data are available online for this figure.

© 2019 The Authors

A

+MS-275 +NaASO2 / -MS-275 -NaASO2	+MS-275 -NaASO2 / -MS-275 -NaASO2	+MS-275 +NaASO2 / -MS-275 +NaASO2	+MS-275 -NaASO2 / -MS-275 +NaASO2	
0.95	NA	0.10	NA	ATXN2
−0.38	0.51	−0.61	−2.26	ATXN2L
0.12	NA	0.57	0.00	DDX1
2.03	0.55	2.27	0.69	DDX3X
0.00	NA	−0.55	−0.98	DDX6
1.14	0.34	1.38	0.85	DHX9
0.25	0.30	1.05	1.19	DYNC1H1
−0.40	0.22	−0.65	0.02	EEF1A1
−0.32	1.44	NA	NA	EEF1A2
0.16	NA	−0.36	−0.61	EEF1B2
0.52	NA	−0.51	−1.07	EEF1D
−0.34	NA	−0.12	1.08	EEF1E1
0.00	0.00	−0.36	−0.61	EEF1G
−0.14	−0.28	−0.57	−0.74	EEF2
1.52	NA	2.97	NA	EIF2A
−0.27	0.12	NA	1.70	EIF2B2
−1.05	−0.60	−0.50	0.46	EIF2B3
NA	0.30	NA	0.65	EIF2B4
−0.62	NA	0.76	NA	EIF2B5
1.10	NA	NA	NA	EIF2D
1.20	−1.81	0.70	−1.26	EIF2S1
0.63	NA	0.43	NA	EIF2S2
0.91	−0.95	0.20	NA	EIF2S3
−0.25	NA	−0.24	0.14	EIF3A
−0.21	−0.11	0.06	−0.04	EIF3B
0.14	−3.81	0.17	−0.90	EIF3C
0.34	0.24	0.41	0.20	EIF3D
−0.13	−0.19	0.00	0.12	EIF3E
−0.59	0.00	0.05	0.20	EIF3F
−0.40	−0.13	−0.27	0.43	EIF3G
−0.09	−0.16	−0.36	−0.48	EIF3H
0.40	−0.22	0.00	−0.16	EIF3I
NA	NA	0.32	NA	EIF3J
NA	NA	−0.61	−3.26	EIF3K
−0.05	0.22	0.13	−0.11	EIF3L
−0.18	NA	0.17	−1.11	EIF3M
−0.13	−0.38	−0.55	−0.94	EIF4A1
1.46	0.15	−0.63	−2.61	EIF4A3
NA	0.65	NA	NA	EIF4B
−0.98	NA	−0.07	−0.02	EIF4E2
1.14	0.42	0.08	−0.86	EIF4G1
0.96	0.64	0.50	−0.82	EIF4G2
NA	NA	−0.04	NA	EIF4G3
1.83	NA	0.25	−2.61	EIF5A
0.37	0.08	1.14	NA	EIF5B
0.81	NA	−0.05	−1.90	EIF6
1.06	0.26	−0.04	NA	ELAVL1
NA	NA	1.14	NA	FXR1
1.68	0.93	1.39	NA	FXR2
−0.03	−0.32	0.46	0.10	G3BP1
0.13	NA	0.34	0.26	G3BP2
−1.02	−0.32	NA	0.50	GIGYF2
1.17	6.11	1.21	1.26	H1F0
1.24	1.50	1.35	2.07	H2AFY
1.72	NA	1.78	1.78	H2AFZ
2.38	2.75	1.96	2.26	HIST1H1B
2.09	7.87	2.11	1.72	HIST1H1D
1.16	1.76	1.21	1.57	HIST1H2BK
1.82	2.45	1.21	2.50	HIST1H4A
1.04	NA	0.71	−0.64	IGF2BP1
NA	NA	−1.05	NA	KHSRP
NA	NA	0.80	NA	KLC1
0.10	NA	−0.61	−0.48	MBNL1
0.91	NA	0.85	−0.42	PABPC1
−0.05	NA	0.48	1.25	PUM1
1.11	0.24	1.67	NA	RBM4
2.50	1.17	3.31	3.23	YBX1

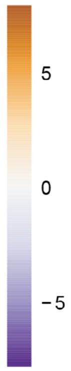

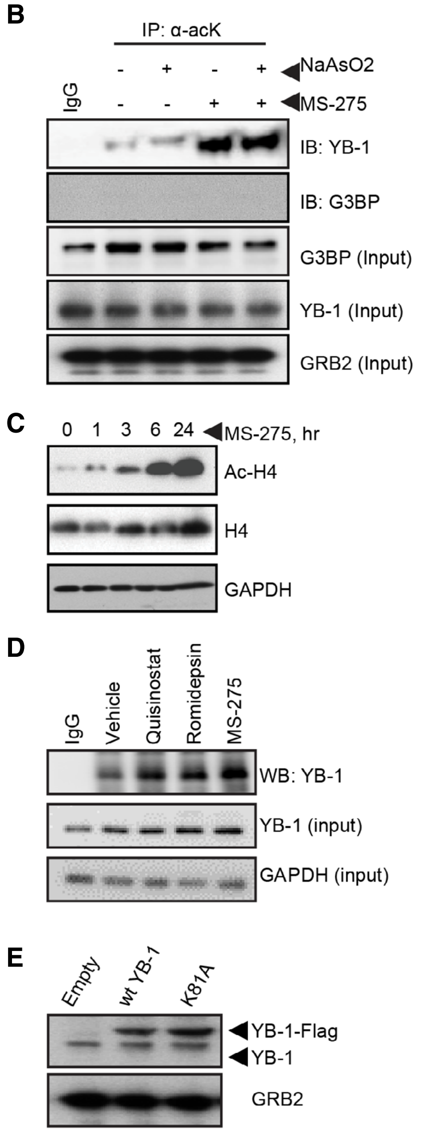

Figure EV2.

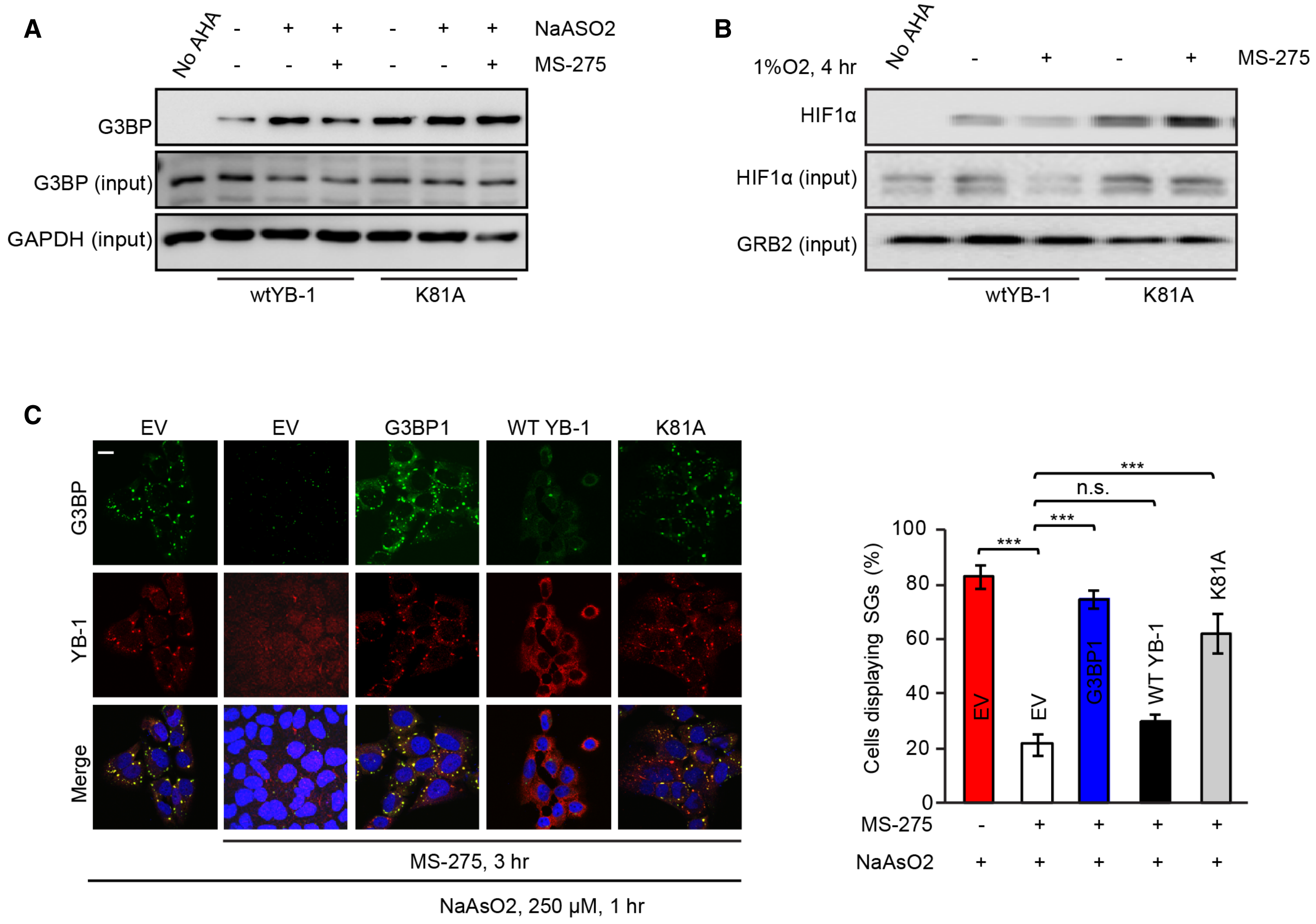

Figure EV3. MS-275-mediated downregulation of YB-1 targets.

A Acutely synthesized G3BP in CHLA-10 cells expressing wtYB-1-FLAG or YB-1-K81A-FLAG. Cells were treated +/− MS-275 (1 μM, 2 h). Then, cells were methionine starved and then pulsed with AHA and $NaAsO_2$ (100 μM), with continuation of MS-275 treatment for 1 h. Acutely synthesized G3BP was identified by immunoblotting with G3BP antibodies (top blot) and compared to total G3BP levels (middle blot). Total GAPDH was used as a loading control (lower blot).

B Acutely synthesized HIF1α in CHLA-10 cells expressing wtYB-1-FLAG or YB-1-K81A-FLAG. Cells were methionine starved and then treated with +/− MS-275 (1 μM) along with AHA for 4 h under hypoxia (1% O_2). Acutely synthesized HIF1α was identified by immunoblotting with HIF1α antibodies (top blot) and compared to total HIF1α levels (middle blot). Total GRB2 was used as a loading control (lower blot).

C Left panel: U2OS-expressing FLAG-tagged empty vector (EV) or the indicated vectors; G3BP1, wtYB-1, or YB-1 K81A mutant, respectively, were treated with MS-275 and $NaAsO_2$ (250 μM), prior to fixation and processing for IF and SG detection. Scale bars = 10 μm. Right panel: SGs were quantified in 20 high-power fields (HPFs) using unpaired two-tailed Student's *t*-test, and results are represented by bar graphs. Error bars indicate SEM. ***$P < 0.0005$; n.s = non-significant.

Source data are available online for this figure.

 © 2019 The Authors

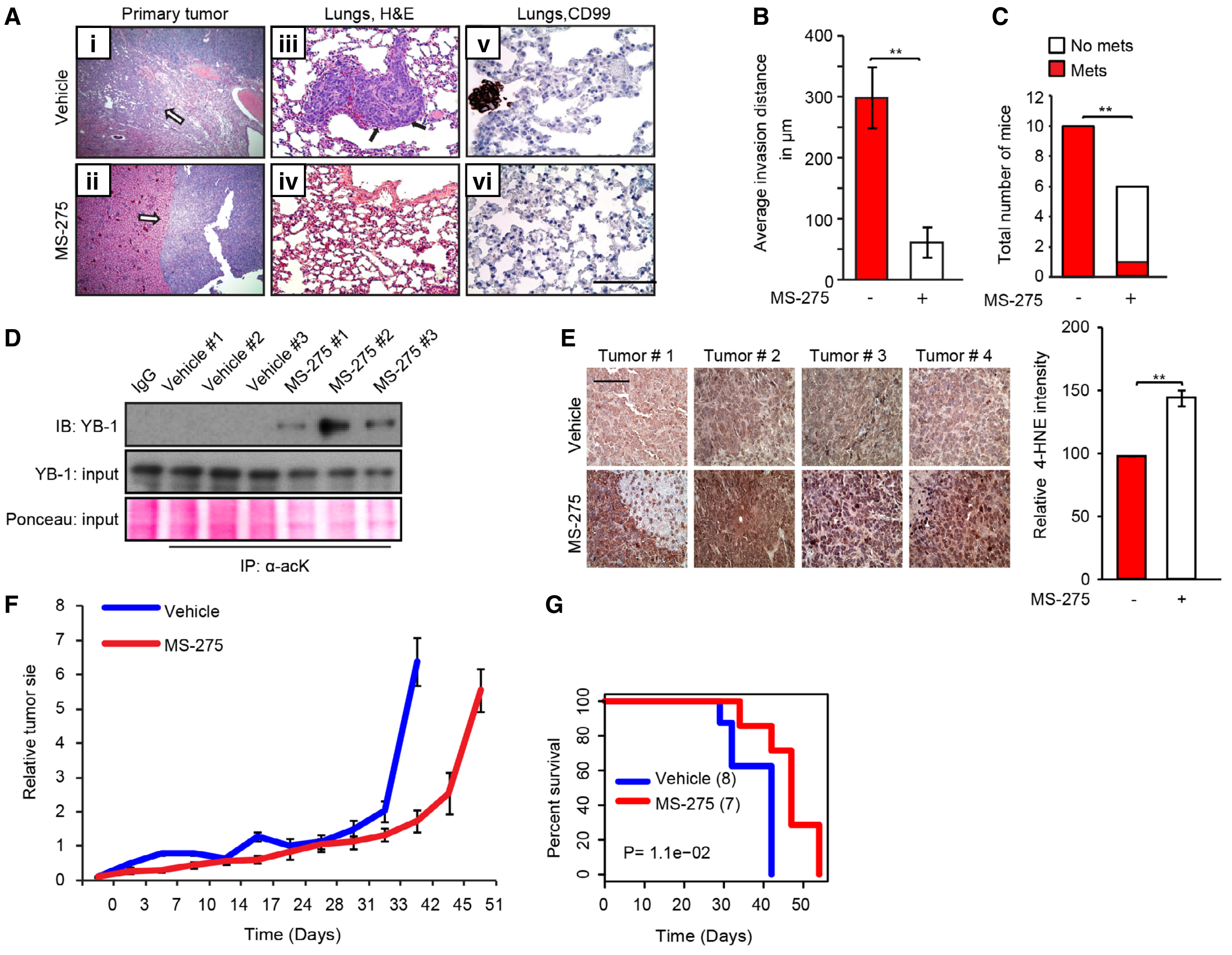

Figure EV4. MS-275 inhibits tumor metastasis *in vivo*.

- A (*i-ii*) H&E-stained representative sections of CHLA-10 xenografts, representing vehicle-treated and MS-275-treated tumors. Arrows show highly invasive growth patterns of vehicle tumor xenografts (*i*) and non-invasive borders of MS-275 treated xenografts (*ii*). (*iii–iv*) H&E staining of metastatic lung lesions (arrows) in mice with renal subcapsular tumor xenografts in the indicated mouse groups. (*v–vi*) Immunohistochemical staining (brown) of the EwS marker CD99 in lung tissues to highlight metastatic lesions.
- B Local invasion in CHLA-10 xenografts was assessed in 5 low power fields per tumor (3 tumors/group) using unpaired two-tailed Student's *t*-test and graphically represented. Error bars indicate SEM.
- C Total number of mice bearing xenografts of the indicated CHLA-10 tumor groups that developed lung metastases, determined using a Fisher's exact test.
- D Analysis of YB-1 acetylation in CHLA-10 tumor lysates −/+ MS-275 as indicated.
- E Left panel: representative IHC images for the oxidative stress marker 4-hydroxynonenal (4-HNE) in renal subcapsular implantation site tumors (tumor #1–4) of the indicated CHLA-10 tumor groups (vehicle and MS-275 treated) using scale bars = 100 µm. Right panel: Quantification of 4-HNE staining intensity in the indicated groups was assessed in 12 HPFs per tumor (4 tumors/group) using ImageJ software, statistically analyzed using unpaired two-tailed Student's *t*-test, and graphically represented. Error bars indicate SEM.
- F Relative tumor sizes in EwS patient-derived xenograft (PDX) model +/− MS-275 treatment (8 mice/group). Tumor measurements were conducted three times/week, and mice were euthanized when humane endpoints were reached. Error bars indicate SEM.
- G Kaplan–Meier survival curves, with *P* values computed with a log-rank test, of ± MS-275 groups of mice showing relative survival in both groups for a total of 55 days starting from the initiation of MS-275 treatment. The numbers of mice are shown in brackets.

Data information: * indicates significant differences between the two groups, $P < 0.05$, $^{**}P < 0.005$.

Source data are available online for this figure.

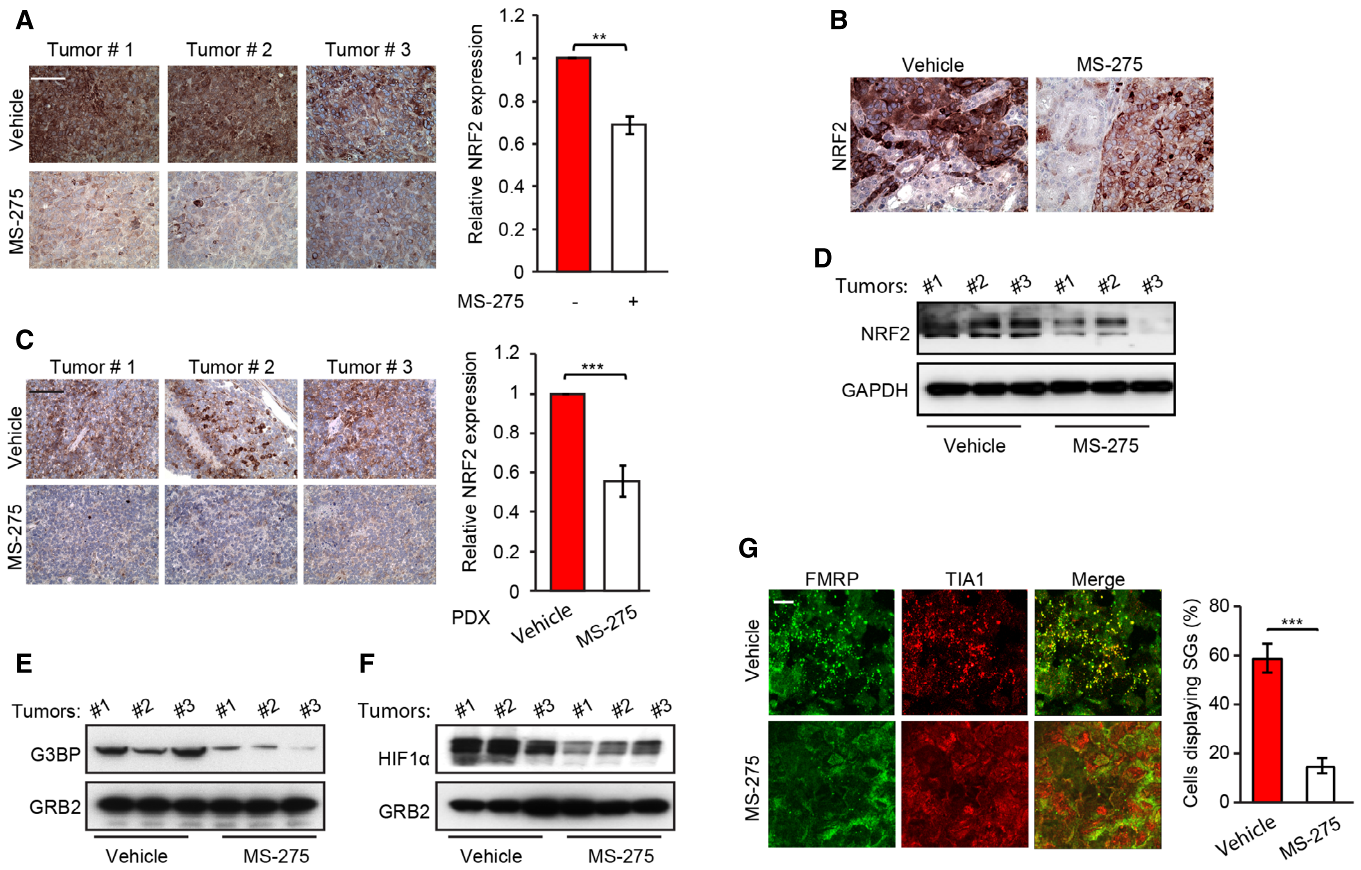

Figure EV5. MS-275-mediated inhibition of NRF2.

A Left panel: IHC of NRF2 in CHLA-10 xenografts from the indicated tumor groups, showing three representative tumors (tumor #1–3) per group. Scale bars = 50 µm. Right panel: Quantitation of staining intensity, normalized to control, was conducted using the color deconvolution plug-in of ImageJ. Error bars indicate SEM for $n = 15$ representative images of three different tumors/group.

B IHC of total NRF2 at the tumor–normal kidney interface in CHLA-10 primary implantation site tumors from the indicated tumor groups.

C Left panel: representative IHC images of NRF2 in tumor xenografts (tumor #1–3) of the indicated EwS IC-pPDX-3 tumor groups (vehicle treated and MS-275 treated). Scale bars = 50 µm. Right panel: Quantification of staining intensity conducted using ImageJ. Error bars indicate SEM for $n = 15$ representative images of three different tumors/group.

D Immunoblot showing NRF2 expression in EwS PDX tumor lysates +/– MS-275 treatment from three independent mouse tumors (vehicle or MS-275 #1-3) per group. GAPDH was used as a loading control.

E Immunoblot showing G3BP expression in CHLA-10 tumor lysates +/– MS-275 treatment from three independent mouse tumors (vehicle or MS-275 #1–3) per group. GRB2 was used as a loading control.

F Immunoblot showing HIF1α expression in CHLA-10 tumor lysates +/– MS-275 treatment from three independent mouse tumors (vehicle or MS-275 #1–3) per group. GRB2 was used as a loading control.

G Left panel: viable tumors areas were cryosectioned and subjected to IF with antibodies to the SG markers, FMRP (green) and TIA1 (red), to identify SGs. Scale bars = 10 µm. Right panel: quantification of stress granules (SGs) in each tumor group shown as a bar graph, in which 15 high-power fields of representative tumor sections from each group ($n = 3$) were used for SG quantification using ImageJ software.

Data information: Unpaired two-tailed Student's *t*-test; $**P < 0.005$; $***P < 0.0005$.
Source data are available online for this figure.

 © 2019 The Authors
