# Supplementary figures and images for: Class I HDAC inhibitors enhance YB‐1 acetylation and oxidative stress to block sarcoma metastasis
Source: EMBO Rep. 2019 Oct 31;20(12):e48375. doi: 10.15252/embr.201948375 (PMC6893361; doi:10.15252/embr.201948375)

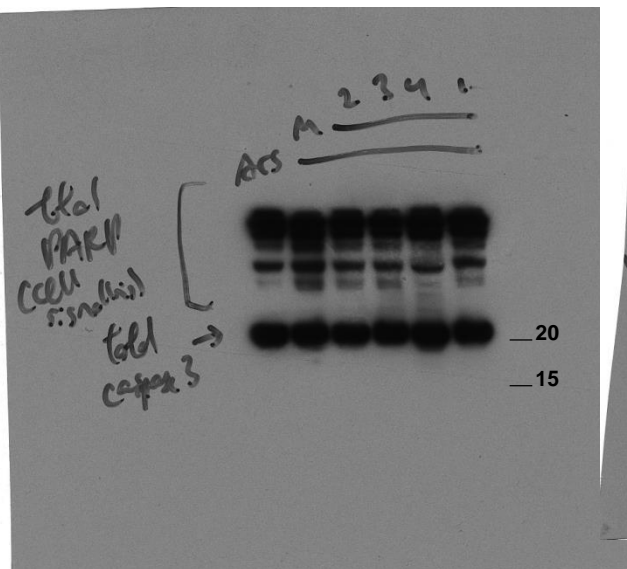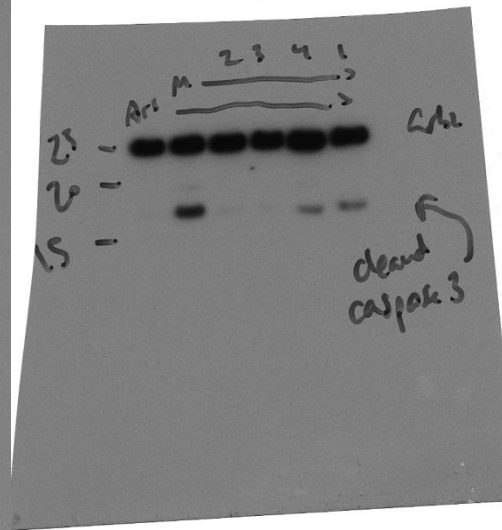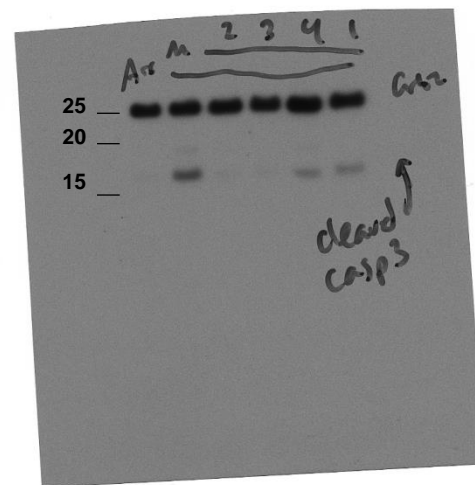

Fig. EV1G

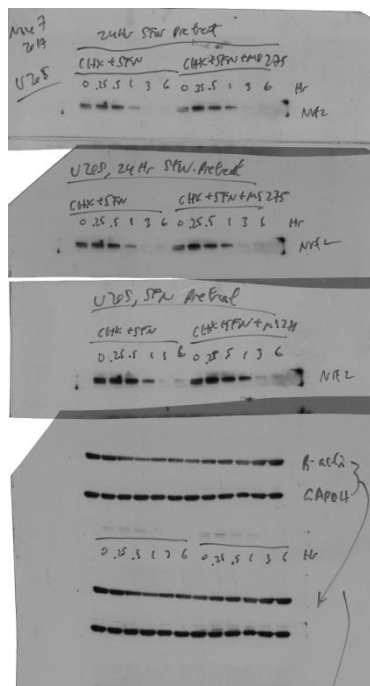

Fig. EV1I

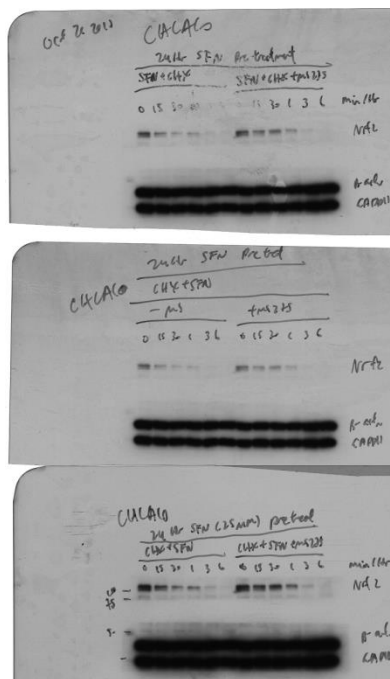

Fig. EV1J

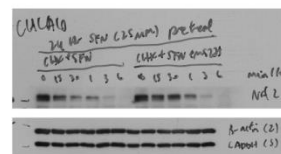

Supplement: Supplementary file 4 — Source Data for Expanded View [file EMBR-20-e48375-s011.zip › Source_Data_for_EV_Figures/SD_Fig_EV1.pdf]

**Fig. EV2B**

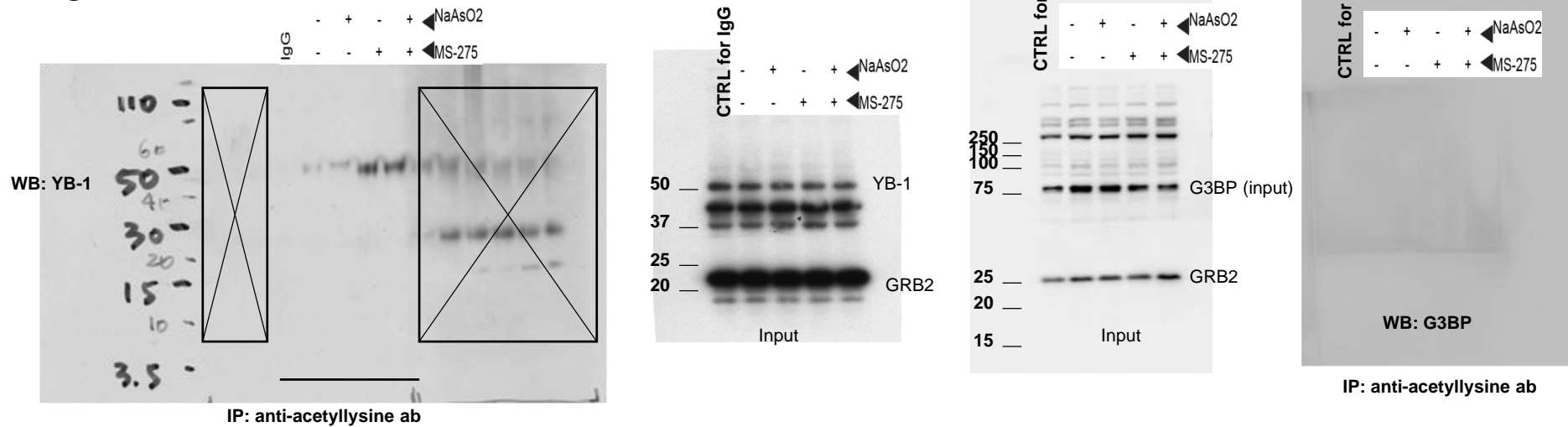

**Fig. EV2C**

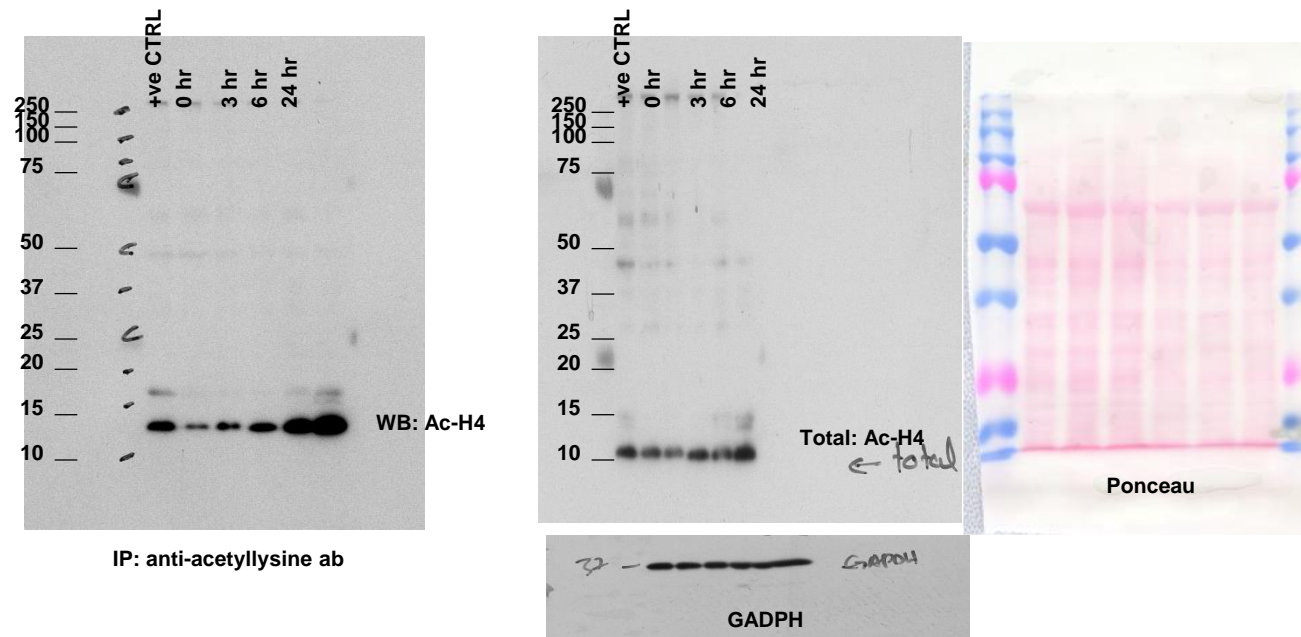

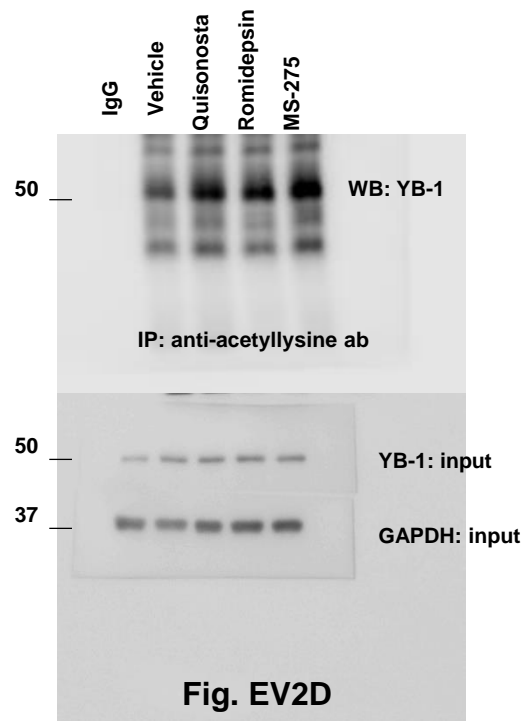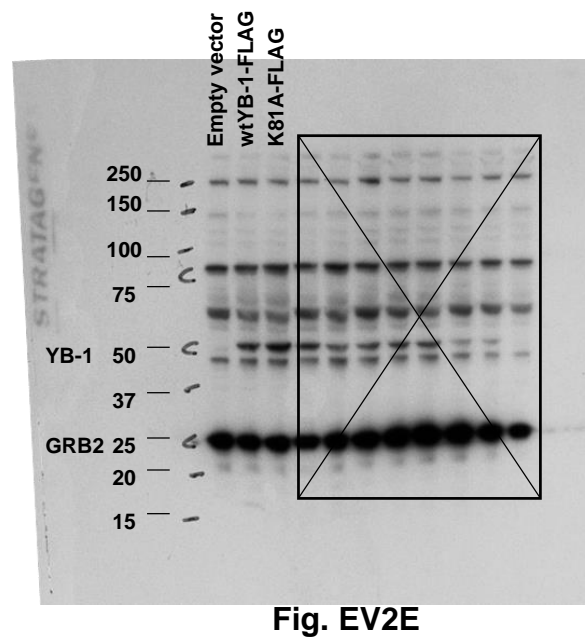

Supplement: Supplementary file 4 — Source Data for Expanded View [file EMBR-20-e48375-s011.zip › Source_Data_for_EV_Figures/SD_Fig_EV2.pdf]

**Fig. EV3A**

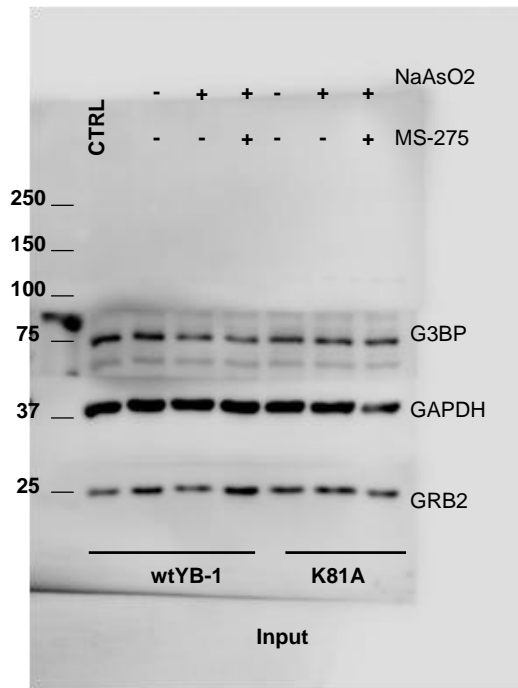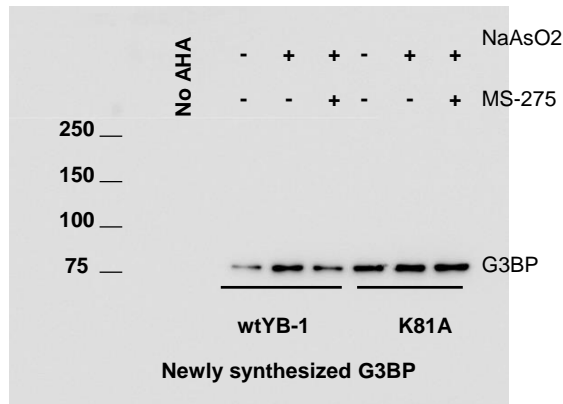

**Fig. EV3B**

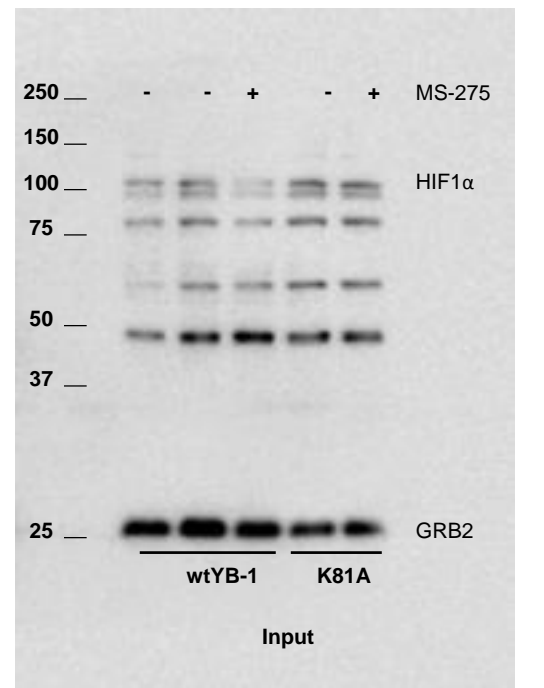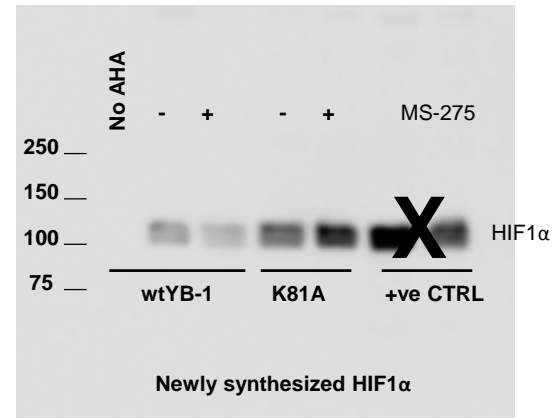

Supplement: Supplementary file 4 — Source Data for Expanded View [file EMBR-20-e48375-s011.zip › Source_Data_for_EV_Figures/SD_Fig_EV3.pdf]

**Fig. EV4F**

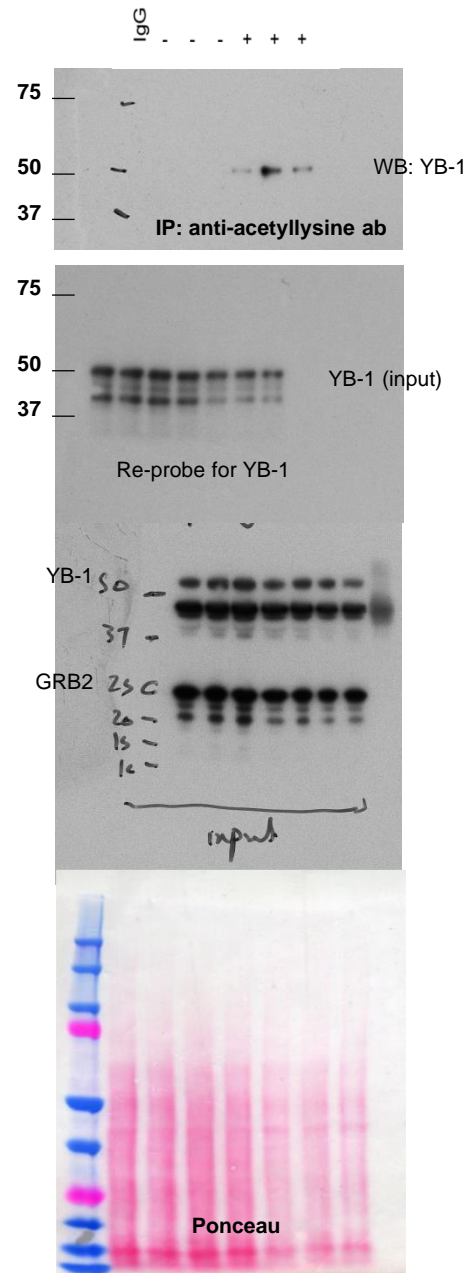

Supplement: Supplementary file 4 — Source Data for Expanded View [file EMBR-20-e48375-s011.zip › Source_Data_for_EV_Figures/SD_Fig_EV4.pdf]

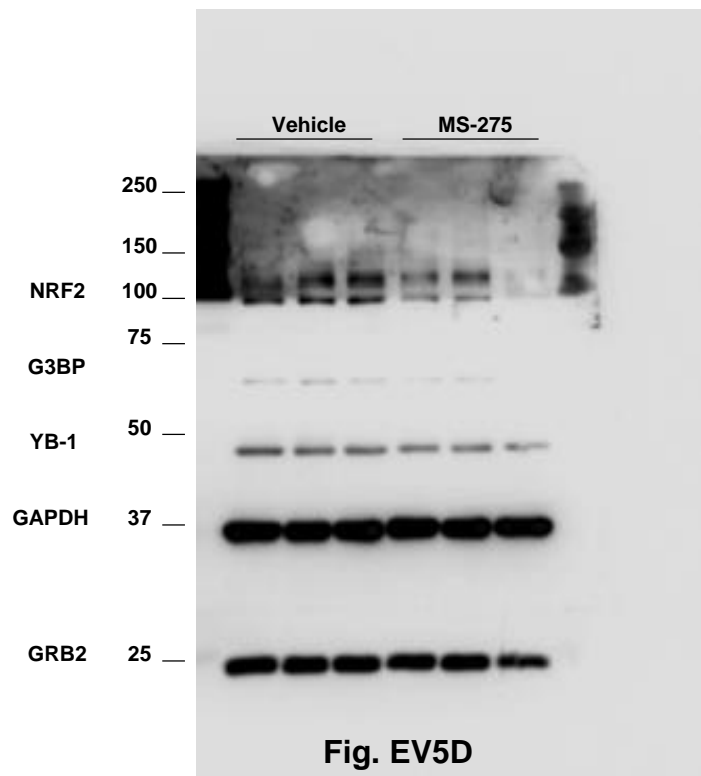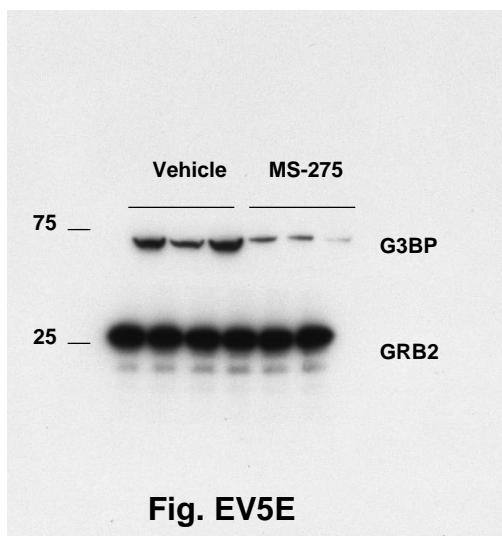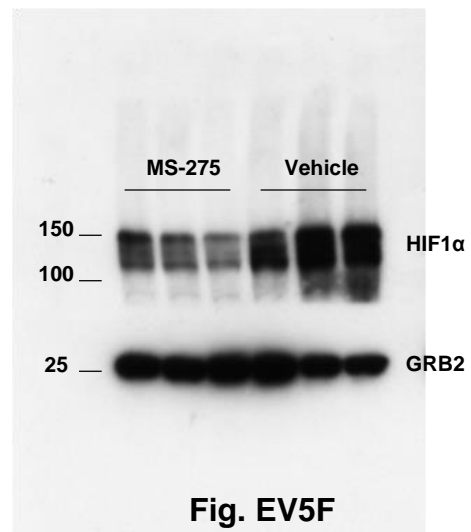

Supplement: Supplementary file 4 — Source Data for Expanded View [file EMBR-20-e48375-s011.zip › Source_Data_for_EV_Figures/SD_Fig_EV5.pdf]

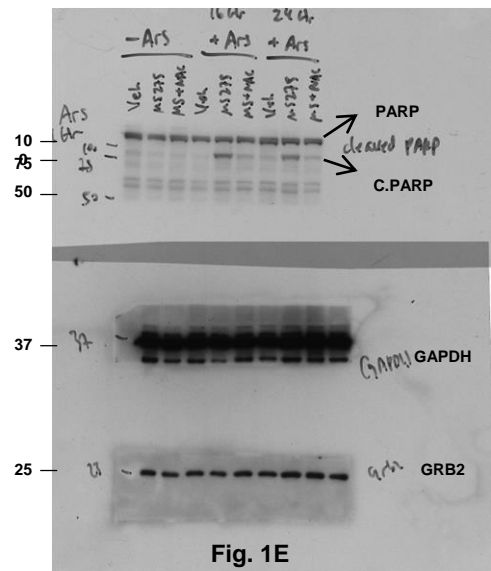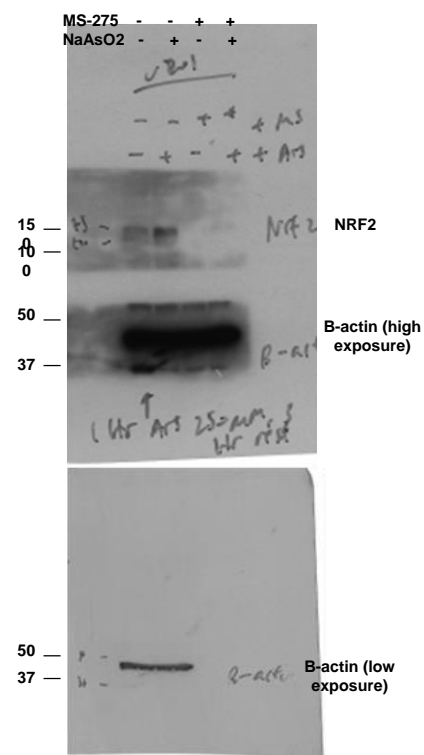

Supplement: Supplementary file 6 — Source Data for Figure 1 [file EMBR-20-e48375-s004.pdf]

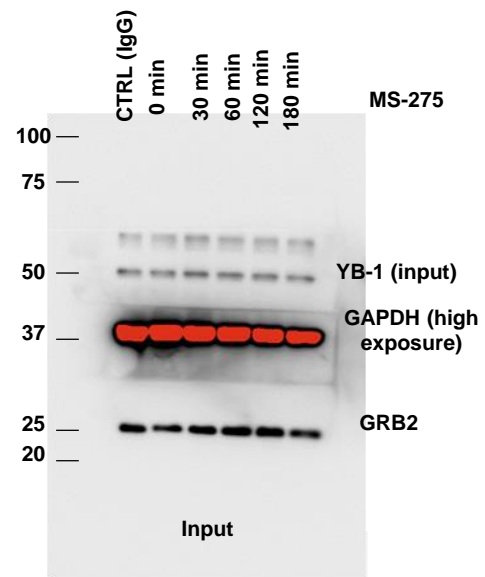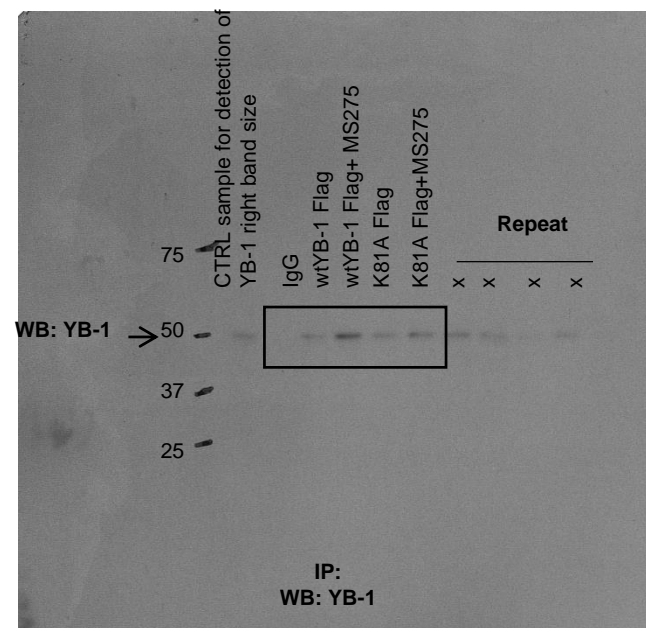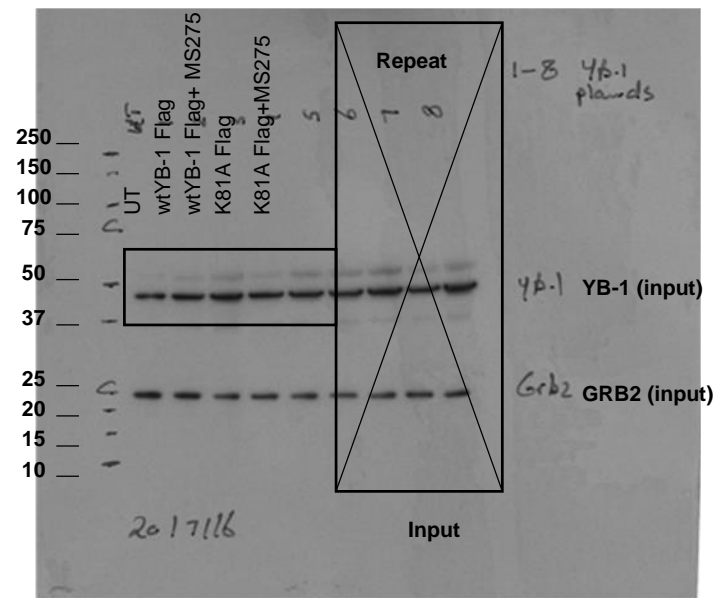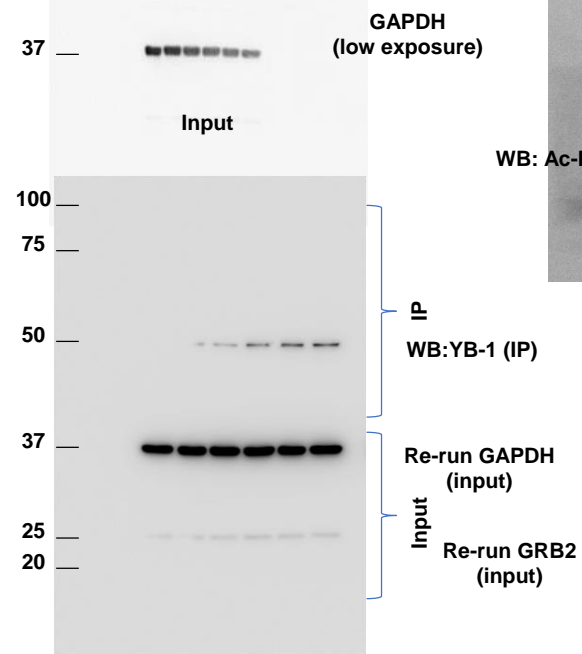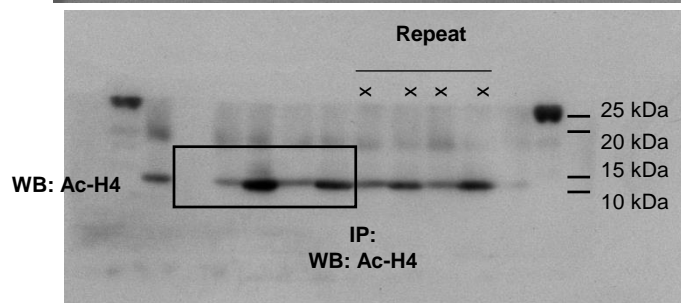

**Fig. 2C**

**Fig. 2B**

Supplement: Supplementary file 7 — Source Data for Figure 2 [file EMBR-20-e48375-s005.pdf]

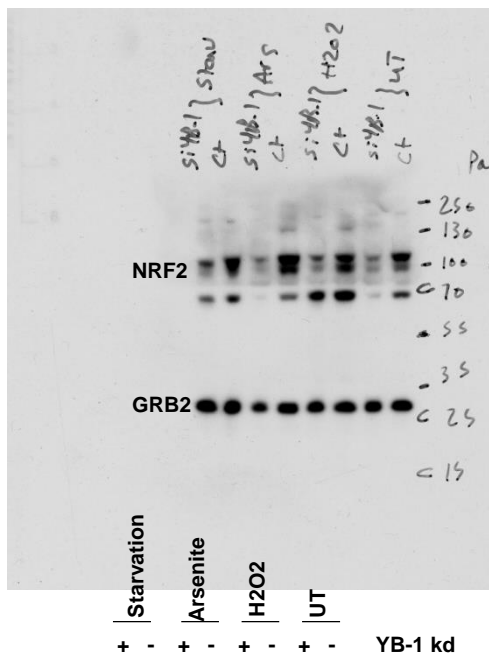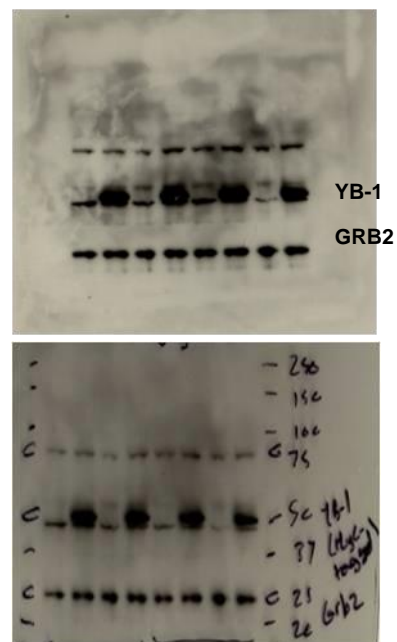

Fig. 3A

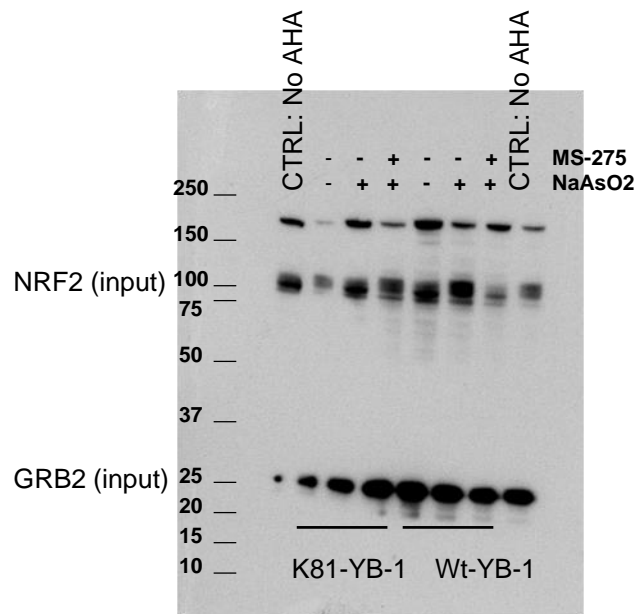

Fig. 3E

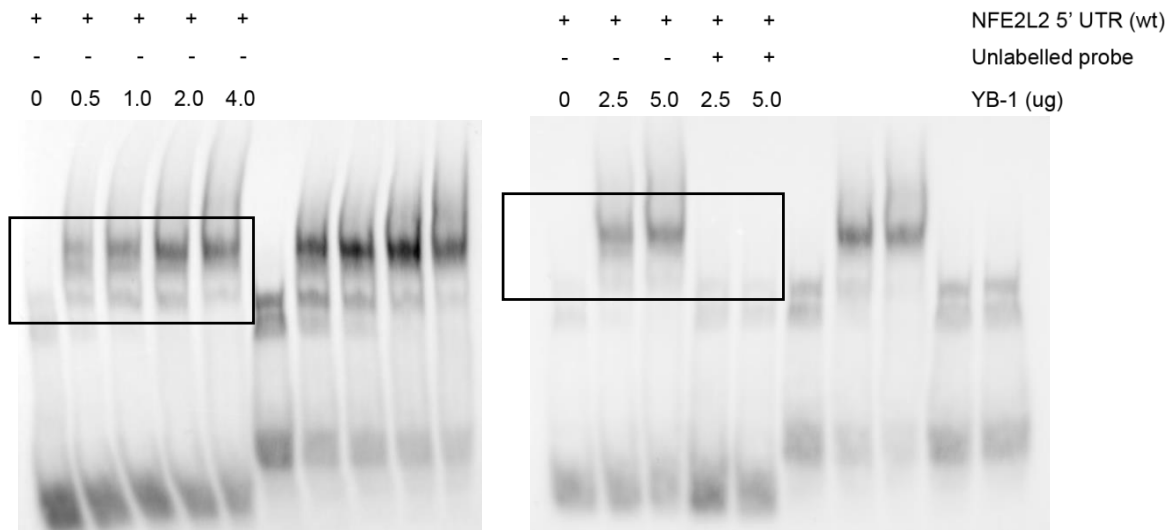

Fig. 3C

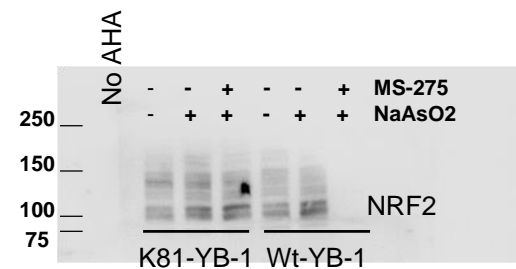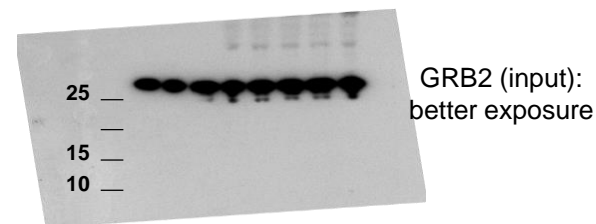

Supplement: Supplementary file 8 — Source Data for Figure 3 [file EMBR-20-e48375-s006.pdf]

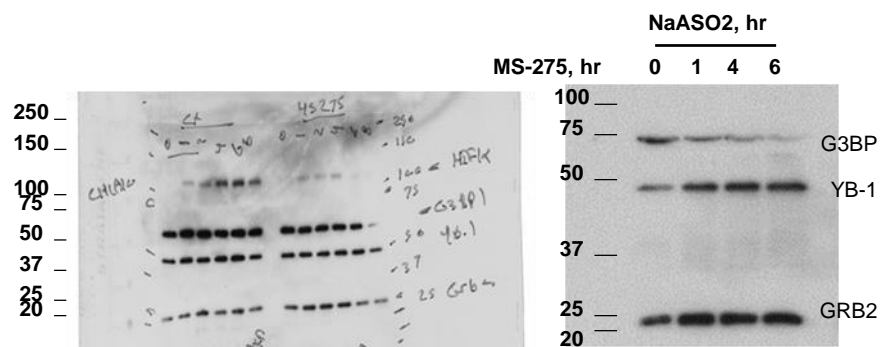

**Fig. 4A**

**Fig. 4B**

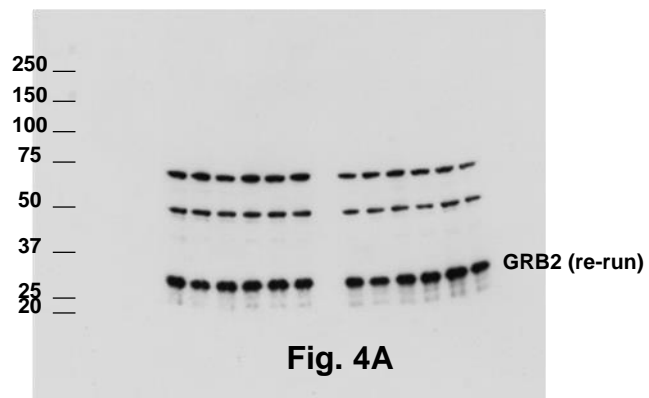

**Fig. 4A**

Supplement: Supplementary file 9 — Source Data for Figure 4 [file EMBR-20-e48375-s007.pdf]

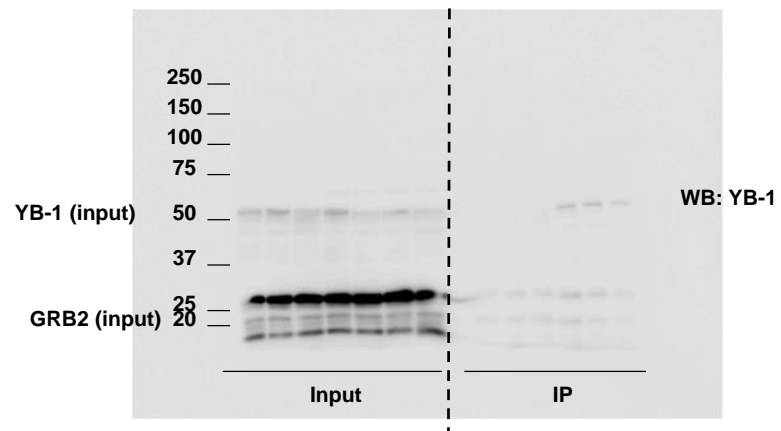

**Fig. 5F**

Supplement: Supplementary file 10 — Source Data for Figure 5 [file EMBR-20-e48375-s008.pdf]

**Fig. 6D**

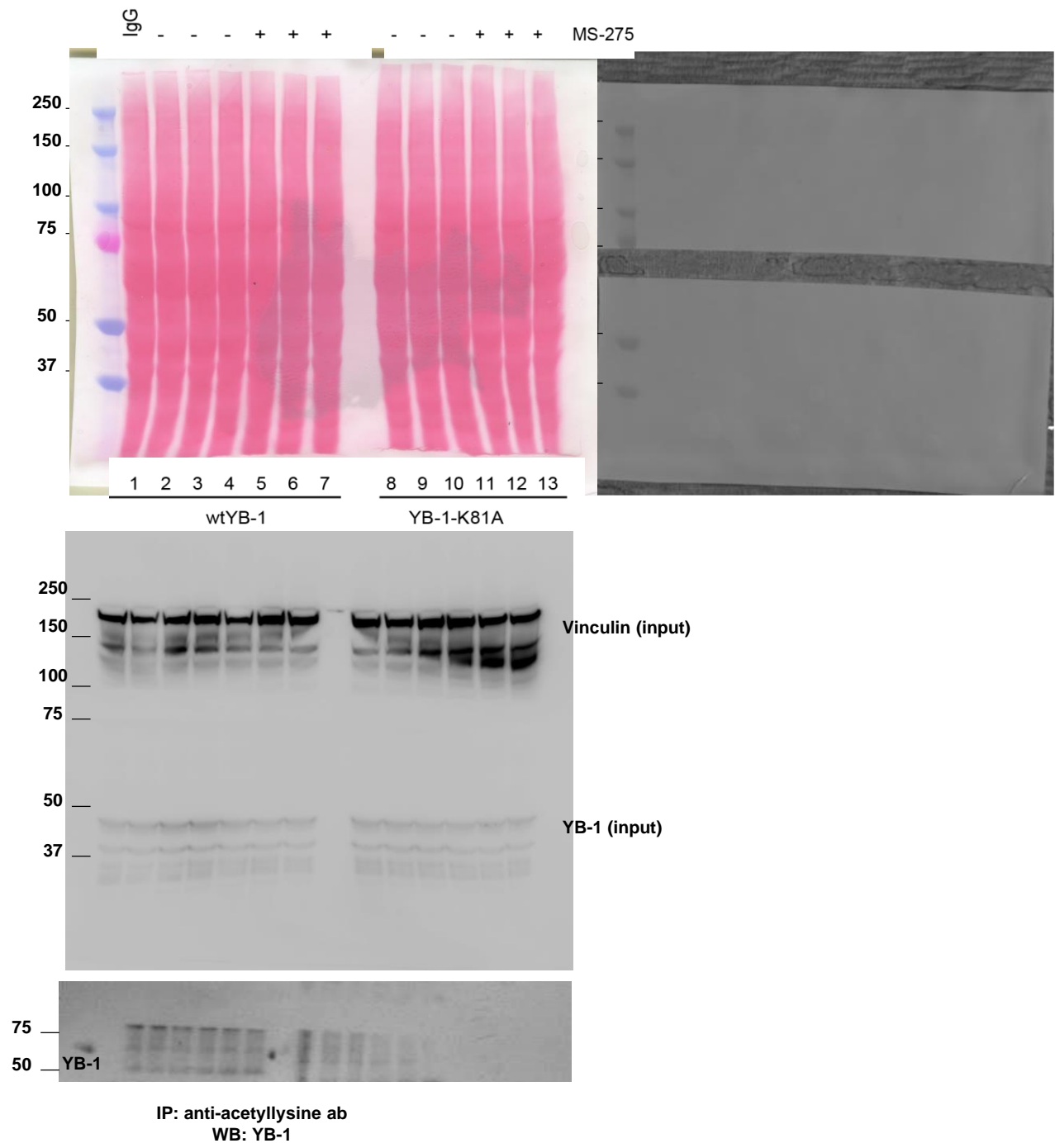

**Fig. 6E**

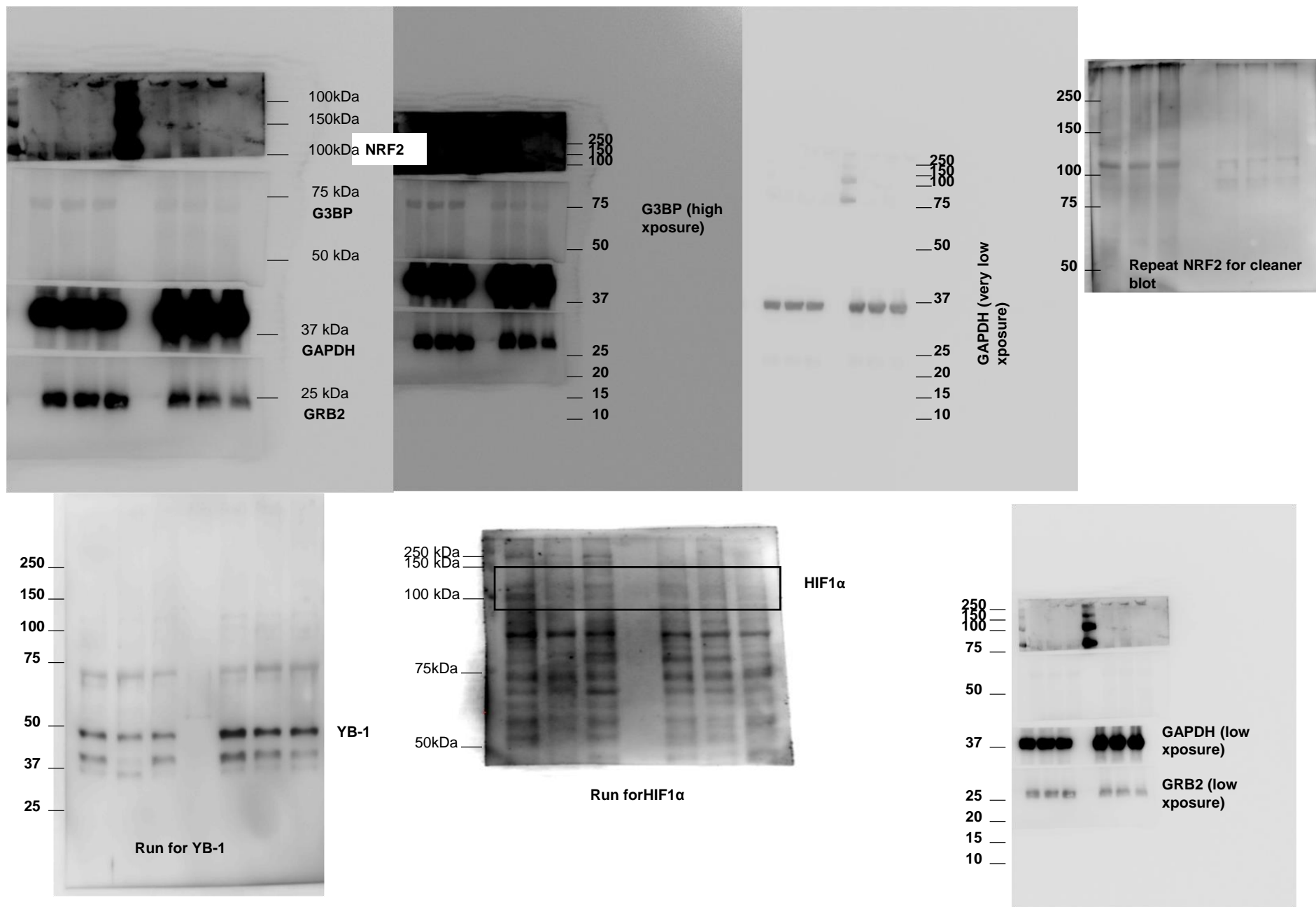

**Fig. 6F**

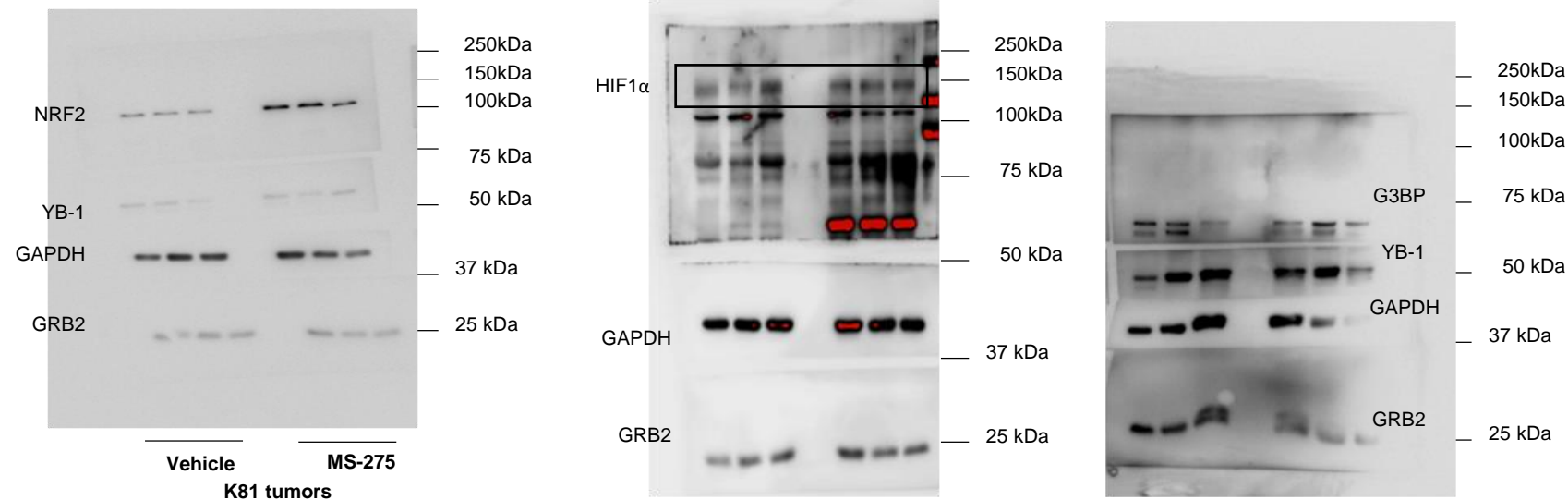

Supplement: Supplementary file 11 — Source Data for Figure 6 [file EMBR-20-e48375-s009.pdf]
