# Supplementary material for: Class I HDAC inhibitors enhance YB‐1 acetylation and oxidative stress to block sarcoma metastasis
Source: EMBO Rep. 2019 Oct 31;20(12):e48375. doi: 10.15252/embr.201948375 (PMC6893361; doi:10.15252/embr.201948375)
Supplement: Supplementary file 12 — Source Data for Figure 8 [file EMBR-20-e48375-s010.pdf]

Fig. 8B

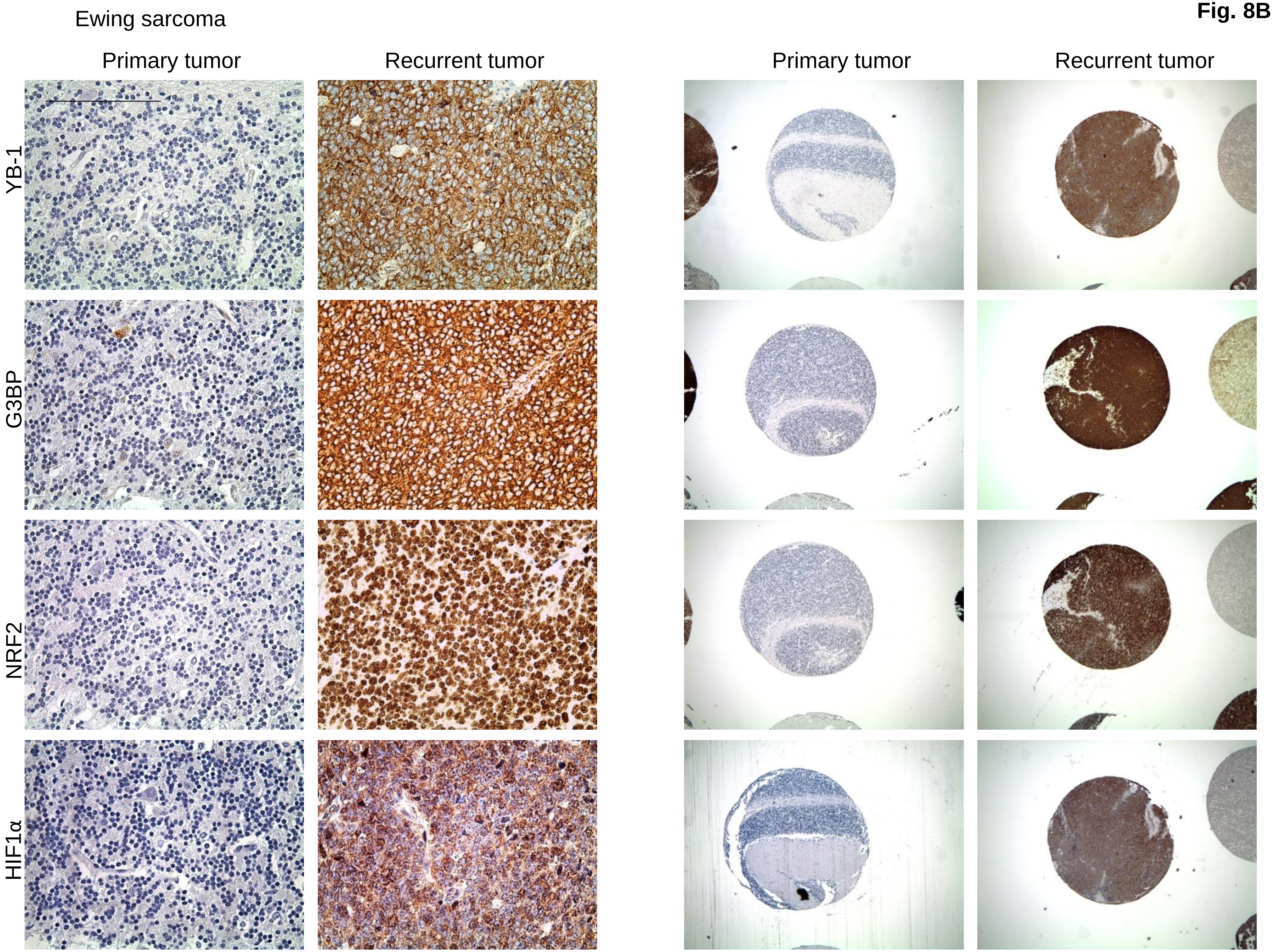
Ewing sarcoma
Primary tumor
Recurrent tumor
Primary tumor
Recurrent tumor
YB-1
G3BP
NRF2
HIF1α

Fig. 8C

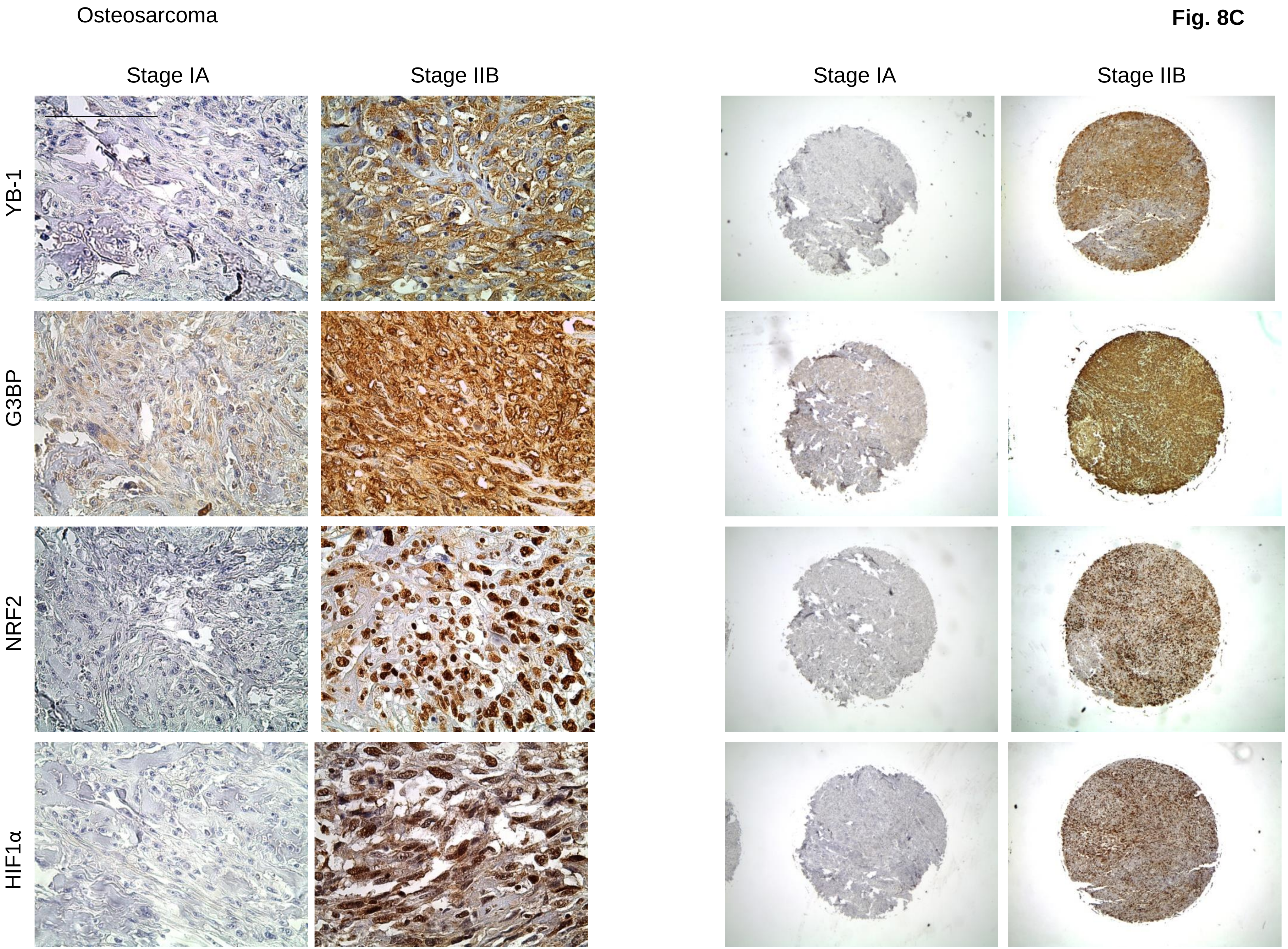
Osteosarcoma
Stage IA
Stage IIB
Stage IA
Stage IIB
YB-1
G3BP
NRF2
HIF1α
