# Supplementary figures and images for: Author Correction: Class I HDAC inhibitors enhance YB-1 acetylation and oxidative stress to block sarcoma metastasis
Source: EMBO Rep. 2025 May 29;26(12):3226–7. doi: 10.1038/s44319-025-00478-6 (PMC12187908; doi:10.1038/s44319-025-00478-6)

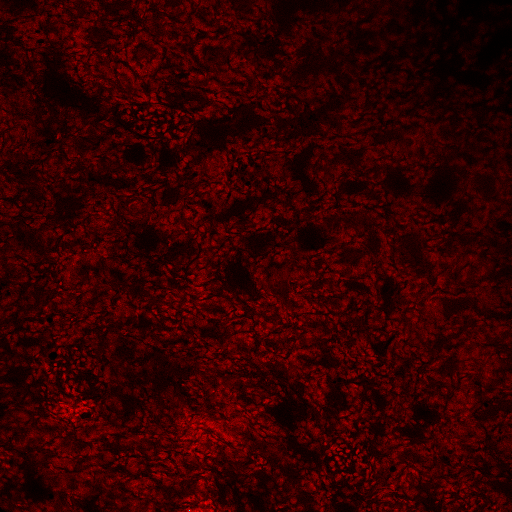

Supplement: Supplementary file 1 — Figure 5A Source Data [file 44319_2025_478_MOESM1_ESM.zip › Vehicle4.tif]

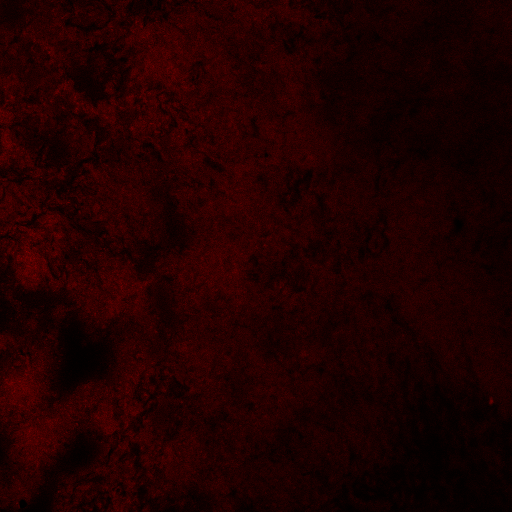

Supplement: Supplementary file 1 — Figure 5A Source Data [file 44319_2025_478_MOESM1_ESM.zip › Vehicle3.tif]

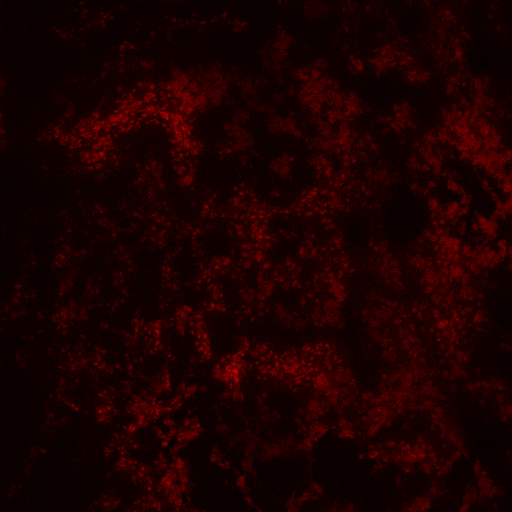

Supplement: Supplementary file 1 — Figure 5A Source Data [file 44319_2025_478_MOESM1_ESM.zip › Vehicle2.tif]

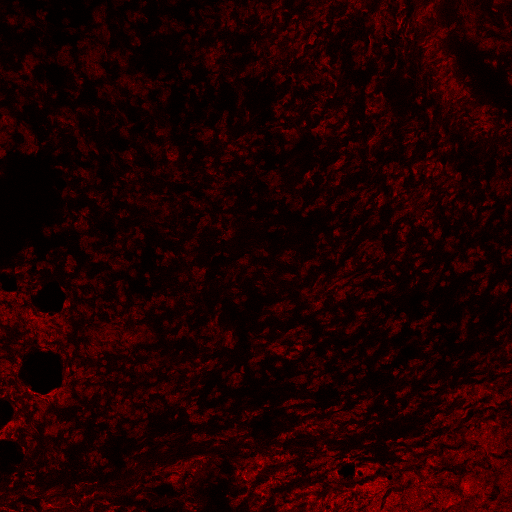

Supplement: Supplementary file 1 — Figure 5A Source Data [file 44319_2025_478_MOESM1_ESM.zip › Vehicle1.tif]

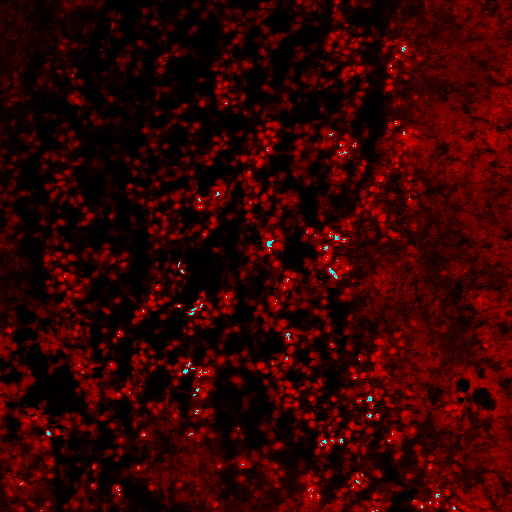

Supplement: Supplementary file 1 — Figure 5A Source Data [file 44319_2025_478_MOESM1_ESM.zip › MS275_4.tif]

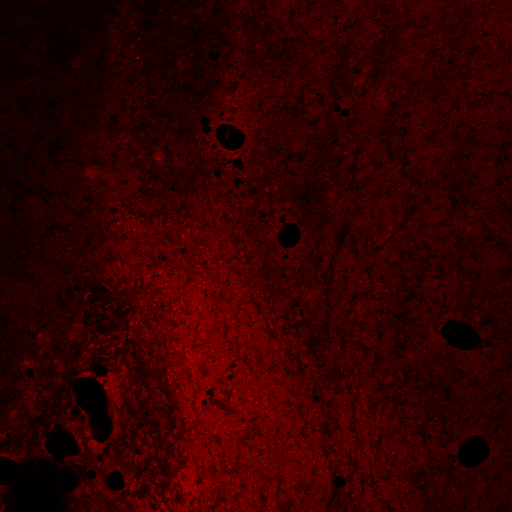

Supplement: Supplementary file 1 — Figure 5A Source Data [file 44319_2025_478_MOESM1_ESM.zip › MS275_3.tif]

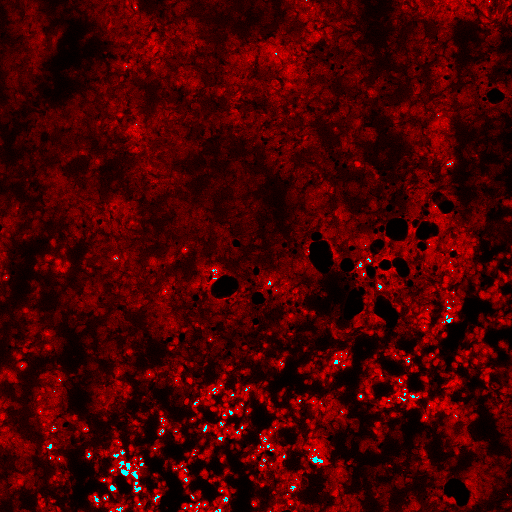

Supplement: Supplementary file 1 — Figure 5A Source Data [file 44319_2025_478_MOESM1_ESM.zip › MS275_2.tif]

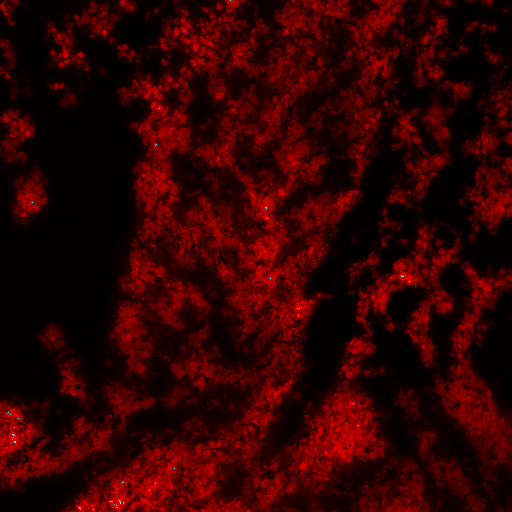

Supplement: Supplementary file 1 — Figure 5A Source Data [file 44319_2025_478_MOESM1_ESM.zip › MS275_1.tif]
